# A Comprehensive Genetic Analysis of Slovenian Families with Multiple Cases of Orofacial Clefts Reveals Novel Variants in the Genes *IRF6*, *GRHL3*, and *TBX22*

**DOI:** 10.3390/ijms24054262

**Published:** 2023-02-21

**Authors:** Lara Slavec, Ksenija Geršak, Andreja Eberlinc, Tinka Hovnik, Luca Lovrečić, Irena Mlinarič-Raščan, Nataša Karas Kuželički

**Affiliations:** 1 Research Unit, Division of Gynaecology and Obstetrics, University Medical Centre Ljubljana, 1000 Ljubljana, Slovenia; 2 Department of Clinical Biochemistry, Faculty of Pharmacy, University of Ljubljana, 1000 Ljubljana, Slovenia; 3 Department of Gynaecology and Obstetrics, Faculty of Medicine, University of Ljubljana, 1000 Ljubljana, Slovenia; 4 Department of Maxillofacial and Oral Surgery, University Medical Centre Ljubljana, 1000 Ljubljana, Slovenia; 5 Clinical Institute for Special Laboratory Diagnostics, University Children’s Hospital, University Medical Centre Ljubljana, 1000 Ljubljana, Slovenia; 6 Institute of Biochemistry and Molecular Genetics, Faculty of Medicine, University of Ljubljana, 1000 Ljubljana, Slovenia; 7 Clinical Institute of Genomic Medicine, University Medical Centre Ljubljana, 1000 Ljubljana, Slovenia

**Keywords:** genetics, family study, non-syndromic orofacial cleft, Van der Woude syndrome, X-linked cleft palate with or without ankyloglossia, *IRF6*, *GRHL3*, *TBX22*, whole exome sequencing

## Abstract

Although the aetiology of non-syndromic orofacial clefts (nsOFCs) is usually multifactorial, syndromic OFCs (syOFCs) are often caused by single mutations in known genes. Some syndromes, e.g., Van der Woude syndrome (VWS1; VWS2) and X-linked cleft palate with or without ankyloglossia (CPX), show only minor clinical signs in addition to OFC and are sometimes difficult to differentiate from nsOFCs. We recruited 34 Slovenian multi-case families with apparent nsOFCs (isolated OFCs or OFCs with minor additional facial signs). First, we examined *IRF6*, *GRHL3*, and *TBX22* by Sanger or whole exome sequencing to identify VWS and CPX families. Next, we examined 72 additional nsOFC genes in the remaining families. Variant validation and co-segregation analysis were performed for each identified variant using Sanger sequencing, real-time quantitative PCR and microarray-based comparative genomic hybridization. We identified six disease-causing variants (three novel) in *IRF6*, *GRHL3*, and *TBX22* in 21% of families with apparent nsOFCs, suggesting that our sequencing approach is useful for distinguishing syOFCs from nsOFCs. The novel variants, a frameshift variant in exon 7 of *IRF6*, a splice-altering variant in *GRHL3*, and a deletion of the coding exons of *TBX22*, indicate VWS1, VWS2, and CPX, respectively. We also identified five rare variants in nsOFC genes in families without VWS or CPX, but they could not be conclusively linked to nsOFC.

## 1. Introduction

Orofacial clefts (OFCs), characterised by the incomplete fusion of certain facial or oral structures, are the most common congenital craniofacial anomalies with global widely varying incidence rates by race and ethnicity. In Slovenia, the average incidence of OFCs is around 1/600 live births (period from 1993 to 2012), which is comparable to other European populations where it ranges from 1/500 to 1/1000 [1,2].

OFCs affect various parts of the oral cavity and face (i.e., palate, alveolus, lip, nose) and, accordingly, are often classified into the following groups: cleft lip; cleft lip and alveolus; cleft lip, alveolus, and palate (CLP); and cleft palate (CP). CP is further divided into complete CP (i.e., hard and soft), soft CP, submucous CP, and bifid uvula. Historically, due to common developmental mechanisms and epidemiological aspects, cleft lip, cleft lip and alveolus, and CLP have often been grouped and studied together as cleft lip with or without cleft palate (CL/P) [3]. However, CP is supposed to have a separate aetiology, but with some overlap [3,4]. Typically, CL/P affects one side (unilateral) or both sides (bilateral) of the lip, alveolus, and/or palate, and appears in varying degrees of severity.

Most OFCs (approximately 70%) are isolated or non-syndromic (nsOFCs) and occur without other structural and/or functional abnormalities, whereas the remaining 30% of OFCs occur as part of various syndromes (syOFCs), caused by single-gene mutations (i.e., Mendelian inheritance), chromosomal aberrations, or teratogenic factors [5,6]. Most common syOFCs include 22q11.2 deletion syndrome (i.e., DiGeorge or velocardiofacial syndrome), Van der Woude syndrome (VWS), and Pierre Robin sequence (PRS) [7]. Many syndromes in which clefting is a major feature have clearly noticeable phenotypes and are easily diagnosed. Nevertheless, there are some syndromes (e.g., X-linked cleft palate with or without ankyloglossia (CPX), PRS, and VWS) where the clinical signs, apart from OFC, are minor or sometimes even unrecognizable, making some syOFCs difficult to distinguish from nsOFCs.

Van der Woude syndrome is one of the most common forms of syOFCs, representing 2% of all OFC cases [8]. The genetic cause can be identified in 75% of VWS cases, with mutations in *IRF6* (VWS1; MIM#119300) in approximately 70% of cases and mutations in *GRHL3* (VWS2; MIM#606713) in the remaining 5% of cases [9,10]. This autosomal dominant syndrome is inherited with high penetrance but variable phenotypic expression [11]. In addition to OFC, VWS is characterized by congenital lower lip pits and in some cases hypodontia [12]. Interestingly, CP and CL/P may both occur in a single VWS family, which is rare in families with nsOFCs [13]. Further, whereas the lip pit phenotype in VWS patients varies from a single, barely visible elevation/depression to pronounced bilateral lower lip pits, 15% of patients lack lip pits altogether [11,12]. Interestingly, deleterious variants in *IRF6* and *GRHL3* were also found in nsOFCs [14,15] in addition to VWS.

X-linked cleft palate with or without ankyloglossia (MIM#303400) is a rare disorder with a semidominant X-linked inheritance of mutations in *TBX22* [16]. It is characterised by a CP phenotype that is most often present in males and ranges from a high-arched palate, bifid uvula, submucous CP, soft CP, to complete CP [17,18]. The main characteristic that divides CPX from nsOFCs is ankyloglossia that is frequently but not always present in affected males and also in female carriers [18]. Family history or pedigree size are sometimes not informative enough to predict the X-linked mode of inheritance, and the diagnosis of CPX may be overlooked [16,19].

Unlike most syOFCs that have a known genetic cause, the aetiology of nsOFCs is complex, since nsOFCs are considered multifactorial disorders that develop due to interactions between genetic and intrauterine environmental factors. Approximately 20% of nsOFC patients come from multi-case families [20]. Individuals with nsOFCs have a significantly increased risk of recurrence in their first-degree relatives (parents, siblings, and offspring) [21]. Moreover, phenotype concordance is 40–60% in monozygotic twins, whereas it is only 3–5% in dizygotic twins, suggesting a significant genetic component in the aetiology of nsOFCs [22,23]. The identification of genetic risk factors for nsOFCs is challenging. To date, many approaches have been used to find candidate regions or genes associated with nsOFCs: cytogenetic studies, linkage analyses, candidate gene association studies (i.e., family- and population-based studies), direct sequencing studies of candidate genes, genome-wide association studies, and studies on animal models [24]. Moreover, in recent years, next-generation sequencing methods, in particular whole exome sequencing (WES), have been increasingly used to determine the genetics of nsOFCs [25,26].

It is a widely accepted hypothesis that complex diseases such as nsOFCs arise from the accumulation of disease-causing variants with relatively high population frequencies (minor allele frequency (MAF) > 5%) [27,28]; however, studies that used different genetic approaches to confirm this hypothesis (i.e., association studies), have explained only a small fraction of heritability of nsOFCs [28,29]. On the other hand, some studies have successfully used WES to identify rare deleterious variants in multi-case families with nsOFCs [30,31,32,33,34,35,36].

In this study, we present the first comprehensive analysis of genetic risk factors for OFCs in the Slovenian population alongside our aim to establish the best diagnostic approach to distinguish between nsOFCs and syOFCs in a cohort of phenotypes resembling nsOFCs and to evaluate a diagnostic gene panel for nsOFCs. A total of 34 Slovenian families with multiple cases of apparent nsOFCs (isolated OFCs or OFCs with minor additional facial signs) were included in the study. Our stepwise diagnostic approach initially examined only three genes implicated in VWS and CPX (i.e., *IRF6*, *GRHL3*, and *TBX22*) using WES and Sanger sequencing. To further determine genetic risk factors for OFCs in Slovenian multi-case families, we later examined 72 additional genes using WES.

Utilizing a two-step diagnostic approach enabled us to differentiate between syOFC cases and nsOFC cases. However, the gene panel was not as informative in families with nsOFCs. We were able to identify the genetic cause of OFCs in 21% of families as we discovered three novel genetic variants causing VWS1, VWS2, and CPX.

## 2. Results

### 2.1. Sequence Analysis of IRF6, GRHL3, and TBX22 to Identify Families with Syndromic Forms of Orofacial Clefts

We examined two genes implicated in VWS, *IRF6* and *GRHL3*, and the gene implicated in CPX, *TBX22*, in 34 multi-case families with apparent nsOFCs (isolated OFCs or OFCs with minor additional facial signs). We identified causal variants confirming VWS in 6 families and CPX in 1 family.

#### 2.1.1. One Novel and Three Previously Described Variants in *IRF6* Confirm VWS1 Diagnosis in Five Families

**Figure 1:**
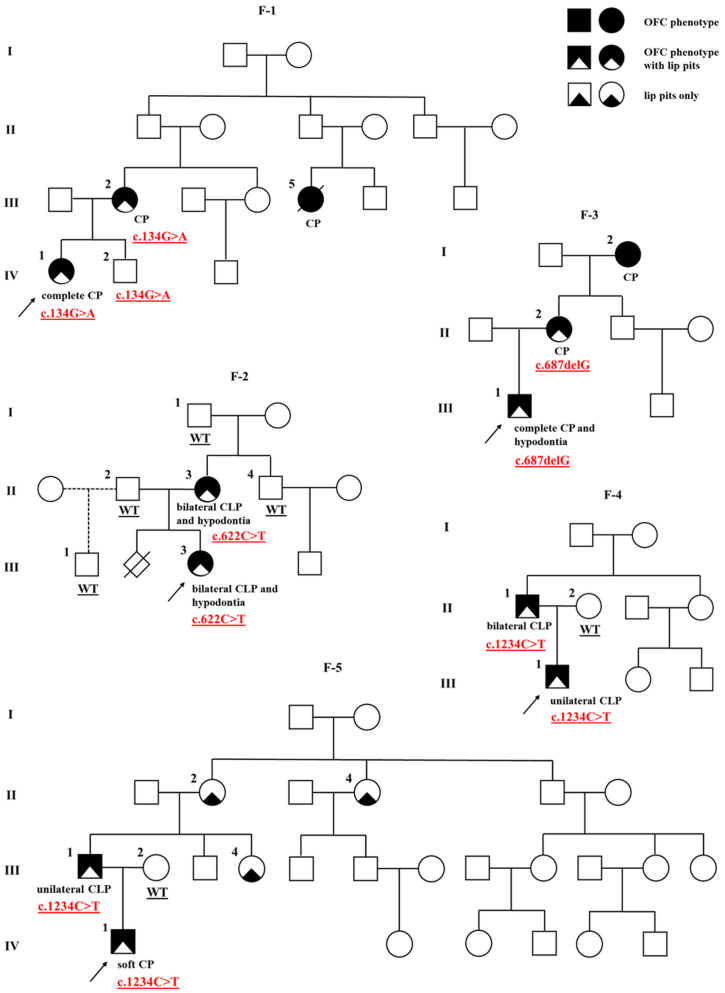
Pedigrees of the five Van der Woude syndrome families (F-1 to F-5) with *IRF6* mutations. The proband is marked with an arrowhead. Genotype is given underneath each genetically tested individual. Roman numerals stand for the generations, while Arabic numerals stand for the individual members of the family. OFC, orofacial cleft; CLP, cleft lip, alveolus and palate; CP, cleft palate.

In total, 7 of the 34 multi-case families with apparent nsOFCs had at least one member with lip pits, suggesting the diagnosis of VWS. We detected one novel and three previously described heterozygous disease-causing variants in *IRF6* (Table 1, Figure 1 and Figure S1) in 5 of the 7 families with suspected VWS. One frameshift, one missense, and two nonsense variants were located in different exons of *IRF6* (3, 6, 7, or 9). To the best of our knowledge, the novel variant, a frameshift variant in exon 7, has not yet been described in the literature, in HGMD Professional 2022.2, or in the ClinVar database. No disease-causing variants in *IRF6* were identified in families with nsOFCs. *IRF6* is intolerant for loss-of-function (LoF) variants (pLI = 1) and shows a degree of intolerance to missense variants (Z = 2.74) as indicated by gnomAD.

##### IRF6:c.134G>A (p.Arg45Gln)

A missense variant in exon 3 of the *IRF6* (NM_006147.4:c.134G>A; rs121434229) was identified in the proband of family 1 (F-1) (Table 1). The substitution was previously detected once in a heterozygous state in gnomAD v2.1.1 (1/251,482 alleles), specifically in one African/African American female. In silico deleteriousness tools for missense substitutions unanimously supported a deleterious effect of the variant on the gene product. The variant is classified as likely pathogenic (PP2, PP3, PP5, PM1, PM2) by ACMG guidelines.

The proband of family 1 is female (F-1; IV-1) with a complete CP and two indistinct lower lip pits, but no other detectable congenital abnormalities. She is the second child in the family, and her older male sibling (F-1; IV-2) is unaffected. The proband’s mother (F-1; III-2) was also born with CP and two lower lip pits. The mother’s cousin (F-1; III-5) apparently had CP and died at the age of 1. Other family members were reportedly unaffected although they were not clinically assessed by a medical professional. Co-segregation analysis has shown that the variant is present in all three examined subjects of the family, the proband, affected mother, and unaffected male sibling (Figure 1).

##### IRF6:c.622C>T (p.Gln208*)

In the proband of family 2 (F-2), we detected a nonsense variant in exon 6 of the *IRF6* (NM_006147.4:c.622C>T) (Table 1). The presence of this variant results in a premature termination codon. It is not present in gnomAD v2.1.1 and is expected to be a loss-of-function variant. It may also activate nonsense-mediated RNA decay (NMD), resulting in haploinsufficiency. The variant is classified as pathogenic (PVS1, PP5, PM2) by ACMG guidelines.

The proband of family 2 is female (F-2; III-3), an only child born with bilateral CLP, two lower lip pits, and dental anomalies, including several missing teeth (hypodontia). The proband’s mother (F-2; II-3) was also born with bilateral CLP, two lower lip pits, and hypodontia. A co-segregation analysis revealed the mother as the affected carrier of the variant. Other family members were reportedly healthy, and the ones available for analysis (F-2; I-1, II-2, II-4, III-1) did not carry the variant (Figure 1).

##### IRF6:c.687delG (p.Lys229Asnfs*13)

In the proband of family 3 (F-3), we identified a novel 1 bp deletion in exon 7 of the *IRF6* (NM_006147.4:c.687delG) (Table 1). This frameshift variant disrupts the reading frame of the sequence and leads to a premature termination codon, which results in the protein product being truncated. This loss-of-function variant may also activate nonsense-mediated RNA decay (NMD), resulting in haploinsufficiency. The variant is not present in gnomAD v2.1.1 and has not been reported before. It is classified as likely pathogenic (PVS1, PM2) using ACMG guidelines.

The proband of family 3 is male (F-3; III-1), an only child born with complete CP, two lower lip pits, and hypodontia (aplasia of several teeth). The proband’s mother (F-3; II-2) was also born with CP and lower lip pits, and the maternal grandmother (F-3; I-2) had CP, but they were not available for further phenotyping. Other family members were reportedly unaffected. Only the proband’s mother was available for co-segregation analysis, and she was found to be the variant carrier (Figure 1).

##### IRF6:c.1234C>T (p.Arg412*)

In the probands of families 4 (F-4) and 5 (F-5), we identified a nonsense variant in the exon 9 of the *IRF6* (NM_006147.4:c.1234C>T; rs1553247595) (Table 1). It leads to the formation of a premature termination codon and is not present in gnomAD v2.1.1. The variant has been shown to reduce IRF6 activity by promoting its degradation on the protein level [44]. Therefore, it is classified as pathogenic (PVS1, PP5, PM2) by ACMG guidelines.

The proband of family 4 is male (F-4; III-1), an only child born with unilateral CLP and two lower lip pits. His father (F-4; II-1) has bilateral CLP and lip pits. The proband’s mother (F-4; II-2) and other family members were reportedly unaffected. In addition to the proband, the variant was detected in the affected father, but not the unaffected mother (Figure 1).

The proband of family 5 is also a male (F-5; IV-1) and an only child. He has soft CP and two lower lip pits. His father (F-5; III-1) was born with unilateral CLP and two lower lip pits, and the father’s sister (F-5; III-4), mother (F-5; II-2) and aunt (F-5; II-4) all have lower lip pits, whereas the proband’s mother (F-5; III-2) is unaffected. Other family members were also reportedly unaffected. The variant was identified in the affected father, whereas samples from other affected members of his family were not available for the analysis (Figure 1).

#### 2.1.2. A Novel Variant in *GRHL3* Suggests VWS2 Diagnosis in One Family

The remaining 2 of the 7 families with suspected VWS did not have disease-causing variants in *IRF6* and no causal variants in *GRHL3*. Interestingly, we identified a splice-altering variant in a family without suspected syOFC. *GRHL3* is intolerant for LoF variants (pLI = 0.99) and shows a small degree of intolerance to missense variants (Z = 1.42) as indicated by gnomAD.

##### GRHL3:c.1285G>T (p.Gly429Cys)

**Figure 2:**
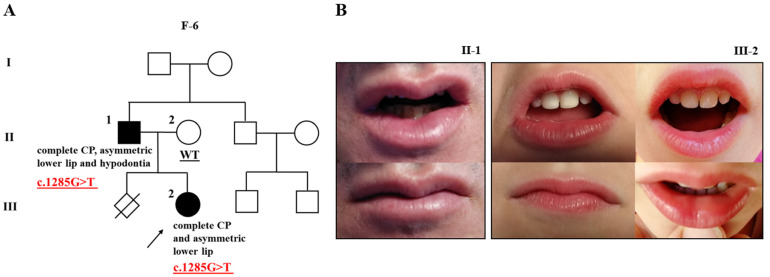
Pedigree of family 6 (F-6) with cleft palate, subtle Van der Woude syndrome sign, and mutation in *GRHL3* (**A**); two affected individuals (F-6; II-1 and III-2) of the family showing an asymmetric lower lip without lip pits (**B**). The proband is marked with an arrowhead. Genotype is given underneath each genetically tested individual. Roman numerals stand for the generations, while Arabic numerals stand for the individual members of the family. CP, cleft palate.

In the proband of family 6 (F-6), we identified a novel donor splice site variant located at the position of the last nucleotide of exon 10 in *GRHL3* (NM_198173.3:c.1285G>T) (Table 1, Figure 2 and Figure S1). This variant is not present in gnomAD v2.1.1, is not listed in dbSNP154, and has not yet been reported in association with VWS. In silico splice site prediction tools unanimously supported a deleterious effect of the variant. Moreover, it is predicted to be deleterious by MutationTester and CADD (score of 35). The tools’ results indicate that the variant most probably affects splicing and is classified as a variant of uncertain significance (VUS) (PM2, PP3) by ACMG guidelines.

The proband of family 6 is female (F-6; III-2), an only child with complete CP. Her father was also born with complete CP (F-6; II-1). Initially, the possibility of VWS was ruled out since they lack lower lip pits. However, a subsequent examination showed an asymmetric lower lip in both the affected father and daughter (Figure 2B), which may subtly indicate the presence of VWS. In addition, the father presents with hypodontia. Other family members were reportedly unaffected. Only the proband’s parents were available for the co-segregation analysis, and the variant was confirmed in the sample of the affected father, but not the unaffected mother (F-6; II-2) (Figure 2A).

#### 2.1.3. A Novel *TBX22* Deletion Reveals a Family with CPX

With the further analysis of the WES data (i.e., computing copy number variations (CNVs)) in families with suspected nsOFC, we have discovered the deletion of *TBX22* on the X-chromosome in the proband of family 7 (F-7). Using the Twist Human Core Exome Plus Kit (Twist Bioscience, San Francisco, USA), we covered only the coding exons of *TBX22* gene (exons 2–9) and established that the deletion is located in the region with the inner start-stop coordinates chrX:g.79,277,769–79,286,610 (hg19) and spans at least 8.8 kb, affecting the entire gene. We did not detect any deletions of the coding regions of adjacent genes or other coding exons on the proband’s X chromosome. Using microarray-based comparative genomic hybridization (array CGH) analysis on the same DNA sample, we further confirmed a hemizygous deletion of 9.91 kb (arr[GRCh37] Xq21.1(79,277,377_79,287,288)x0) encompassing exons 2–9 of the *TBX22* gene (Figure S2). This analysis showed that the non-coding exon 1 of *TBX22* is intact and also revealed the first signal 3.8 kb downstream of the *TBX22* gene, limiting the size of the deletion and confirming that it does not include other genetic material. The identified deletion, encompassing only *TBX22*, has not been reported before and is classified as pathogenic by ACMG standards.

**Figure 3:**
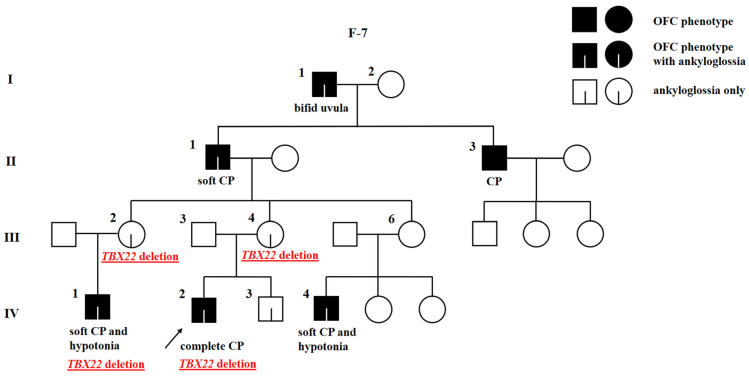
Pedigree of family 7 (F-7) with X-linked cleft palate with or without ankyloglossia and deletion of *TBX22*. The proband is marked with an arrowhead. Genotype is given underneath each genetically tested individual. Roman numerals stand for the generations, while Arabic numerals stand for the individual members of the family. OFC, orofacial cleft; CP, cleft palate.

The proband of family 7 is male (F-7; IV-2), born with complete CP (Figure 3). His brother (F-7; IV-3), father (F-7; III-3), and mother (F-7; III-4) are apparently unaffected. The OFC is inherited through the maternal side. The mother’s grandfather was born with bifid uvula (F-7; I-1), her father (F-7; II-1) with soft CP, and her uncle (F-7; II-3) with an unknown kind of CP. The mother’s two sisters (F-7; III-2, III-6) each have one son with soft CP (F-7; IV-1, IV-4). The family history was reassessed after genetic testing. Ankyloglossia was identified in the proband (F-7; IV-2), his unaffected brother (F-7; IV-3), his mother (F-7; III-4), one of his unaffected aunts (F-7; III-2), both affected cousins (F-7; IV-1, IV-4), his affected grandfather (F-7; II-1), and his affected great-grandfather (F-7; I-1). In some cases, ankyloglossia was corrected immediately after birth or later in life and not recorded in the medical records. Moreover, the family also reported that the proband’s affected cousins (F-7; IV-1, IV-4) had hypotonia. Other family members are reportedly unaffected. The hemizygous loss of all coding exons of *TBX22* detected by WES and array CGH in the proband (F-7; IV-2) was confirmed by real-time quantitative PCR (qPCR). His mother (F-7; III-4) was found to be a carrier, and the variant was also confirmed in his affected cousin (F-7; IV-1) and aunt (F-7; III-2). Other samples were not available for the analysis. The qPCR results are reported in Table S1. The loss of *TBX22* in this family suggest the diagnosis of X-linked cleft palate with or without ankyloglossia. The X-linked inheritance mode does not match with the proband’s great-grandfather’s (F-7; I-1) phenotype. There is no evidence of a consanguinity between his great-grandparents and no history of OFC in his great-grandmother’s (F-7; I-2) family.

### 2.2. Sequence Analysis of Additional 72 Genes in the Families with Apparent Non-Syndromic Orofacial Clefts

Further genetic risk factors for OFCs in Slovenia were determined by examining 72 additional genes in multi-case families lacking disease-causing variants in *IRF6*, *GRHL3,* or *TBX22* or with no VWS or CPX diagnosis (*n* = 27). Thus, we identified 14 rare variants that fit our inclusion criteria:

- five rare variants with inconclusive involvement in OFCs (Table S2);
- nine rare variants that were excluded after co-segregation analysis (Table S3).

The involvement of five rare variants in nsOFCs could not be conclusively determined based on the results of in silico prediction tools, co-segregation analysis, and the literature (Table S2). In the proband of one family with nsOFC, we identified in-frame insertion in *FGFR1* (NM_023110.3:c.396_398dup) and a missense variant in *JAG2* (NM_002226.5:c.3004A>G) in another. Both variants co-segregate with the disease phenotype but are also present in the unaffected siblings of the probands, suggesting that the variant is either not causal or that its penetrance is reduced. In addition, c.3004A>G (*JAG2*) was not predicted to be damaging by the majority of in silico tools used, although it was predicted uncertain by Franklin’s aggregated prediction. The variant in *TBX22* (NM_001109878.2:c.1489G>A) segregates with the disease phenotype in the family but occurs at the end of last exon (exon 9) and is predicted to be benign by the majority of in silico tools (uncertain by Franklin’s aggregated prediction). A co-segregation analysis failed to yield an informative result for the variant in *DLG1* (NM_001366207.1:c.2048-22_2048-4del) due to the absence of the sample from the affected sibling, whereas the unaffected mother does not carry the variant. Finally, the variant in *BMP4* (NM_001202.6:c.272C>G) is unanimously predicted to be deleterious by in silico tools and co-segregates with disease phenotypes in the family, but in the ClinVar database, researchers provided conflicting interpretations of pathogenicity, ranging from uncertain significance to likely benign. We also report nine rare variants that were studied for their involvement in OFCs in our cohort but were excluded after co-segregation analysis because they did not segregate with the OFC phenotype (Table S3).

## 3. Discussion

The present study employed genetic analysis to examine 34 Slovenian families with multiple cases of apparent nsOFCs (isolated OFCs or OFCs with minor additional facial signs) to identify rare disease-causing variants and found 6 deleterious variants in 7 families, 3 of which were novel.

All variants were found in three genes, *IRF6*, *GRHL3*, and *TBX22*, which are involved in the known syndromes, VWS and CPX. In addition, we discovered five rare variants in probands with nsOFCs, where their involvement in the disease could not be conclusively determined.

In five of seven families with suspected VWS (71.4%), we found four heterozygous variants in *IRF6* that are classified as pathogenic or likely pathogenic according to ACMG guidelines. The figure is consistent with previous studies in which *IRF6* variants were detected in approximately 67% of VWS cases [9,45]. In addition, we discovered a heterozygous likely causal splice-altering variant in *GRHL3* in one family with suspected nsOFC, which is classified as VUS according to ACMG guidelines. On subsequent examination of the family, we recognized atypical but identifiable signs of VWS.

*IRF6*, the first gene of interest, has 9 exons, 7 of which are coding (exons 3–9) [46], and they encode a protein with a highly conserved N-terminal DNA-binding domain (helix-turn-helix) (exons 3 and 4) and the less conserved C-terminal protein-binding domain called SMIR (exons 7 and 8) [4,47]. Researchers have identified numerous *IRF6* variants associated with VWS, allowing them to examine their distribution among coding exons [9] and to define the IRF6 domains in which variants are most likely to affect IRF6 function [48]. De Lima et al. showed that deleterious variants in *IRF6* occur significantly more frequently in exons 3, 4, 7, and 9. In addition, they observed frameshift and nonsense variants (protein truncating variants) in all *IRF6* exons of the VWS families, whereas missense variants and in-frame indels are significantly overrepresented in the exons encoding conserved DNA-binding or SMIR domain [9]. Leslie et al. further demonstrated that syndromic features arise from rare variants in the coding sequence of *IRF6* (particularly the DNA-binding domain), because these variants are very rare in controls [48]. The high frequency of protein-truncating variants in VWS [9] and data from functional studies [44] suggest that the cause of VWS is most likely haploinsufficiency of *IRF6*.

In our cohort of VWS families, there were four *IRF6* variants. The missense variant c.134G>A (rs121434229), located in the DNA-binding domain (exon 3), was identified in the affected mother and daughter with complete CP and lower lip pits, and in the unaffected son (F-1). The in silico tools unanimously supported a deleterious effect of the variant, although we noted incomplete penetrance. The variant was previously described in a Japanese VWS family where one patient had CL and lip pits, whereas the father and uncle only had lip pits [37]. These data suggest that this variant is associated with phenotypic variability. A nonsense variant c.622C>T in *IRF6* (exon 6) was found in both affected individuals in one family (F-2), the mother and daughter with bilateral CLP, lower lip pits, and hypodontia. This loss-of-function variant was previously identified in a male Honduran VWS patient with unknown family history who had unilateral CL/P and two lower lip pits [38]. We also identified a novel variant c.687delG, a frameshift deletion located in the SMIR domain (exon 7) of IRF6, which is not present in gnomAD v2.1.1. This loss-of-function variant was confirmed in both mother and son with complete CP and lower lip pits (F-3). Lastly, we identified another nonsense variant (in exon 9) c.1234C>T (rs1553247595) in two families. This loss-of-function variant is located within a CpG dinucleotide and could result from a cytosine methylation/deamination process [9,49]. Phenotypic variability was observed in both families. In the first family (F-4), the phenotype ranges from bilateral CLP and lip pits in the father to unilateral CLP and lip pits in the son, and in the second family (F-5), the father presents with unilateral CLP and lip pits and the son with soft CP and lip pits. The variant is one of the five most common variants in VWS [9], having been identified previously in numerous VWS families with variable phenotypic expressions from Brazil, China, Honduras, northern Europe, Pakistan, and Singapore [4,9,38,39,40,41,42,43]. Observed phenotypic variability and incomplete penetrance are common features of VWS and may be due to stochastic effects and/or genetic modifiers.

In contrast to VWS1, which arises from rare protein-altering *IRF6* variants [4], nsOFCs are significantly associated with common *IRF6* variants in European populations [14,50]. Lately, scientists focused on rare deleterious variants in numerous genes that might explain some heritability of complex nsOFC aetiology [13,25,26,30,31,32,33,34,35,36,45,51]. In the study by Leslie et al. [13], more than 1500 nsOFC families were screened for variants in *IRF6*, and the literature on similar studies was reviewed to determine that rare *IRF6* variants occur in less than 0.5% of probands with nsOFCs. Even though we included only families with multiple cases of nsOFCs, it is not surprising that we were unsuccessful in finding rare *IRF6* variants in our small cohort. This further supports the thesis that rare coding variants are unlikely to play a major role in nsOFCs [13].

Another gene of interest, *GRHL3*, has 10 protein-coding transcripts that differ in both length and exon number. The Ensembl canonical transcript has 16 coding exons [46,52]. *GRHL3* encodes a protein with transactivation (exons 2–3), DNA-binding (exons 6–10), and dimerization (exons 13–16) domains (according to the GRHL3 protein NP_937816.1). In vivo studies suggest that proteins encoded by mutated *GRHL3* cause VWS through a cell-autonomous dominant-negative effect [10]. According to HGMD, variants in *GRHL3* (missense/nonsense variants, splicing substitutions, and small indels) cause either VWS2, non-syndromic cleft palate, or spina bifida. In one of the families with suspected nsOFC (F-6), we identified a novel splice site variant c.1285G>T in exon 10 of *GRHL3* (within the DNA-binding domain). The variant is predicted to alter the donor splice site and is not present in gnomAD v2.1.1. It was detected in both affected individuals, father and daughter, both presenting with complete CP. Subsequent examination revealed a somewhat asymmetric lower lip with elevations in both and hypodontia in the father. The daughter was too young to have permanent teeth and was not available for dental anomaly examination with dental imaging techniques. Other phenotypes that were present in addition to OFC suggested the diagnosis of VWS2 in this family. Two studies identified deleterious variants in close proximity to c.1285G>T. In a patient with non-syndromic cleft palate Eshete et al. [51] identified a dominant-negative missense/splice-site variant c.1282A>C (*GRHL3*), which is three nucleotides upstream of our variant. The presence of lip pits and dental anomalies was not referenced. In addition, Mangold et al. [15] reported a donor splice-altering variant c.1285+2delT (*GRHL3*), located only two nucleotides downstream of the variant reported herein in a nsOFC family with a phenotype highly similar to the one observed in two affected individuals from the present study (F-6). Two half-sisters had a complete CP and a slightly asymmetric lower lip with elevation on the left side resembling lower lip pits, which could be interpreted as a subtle VWS sign. Hypodontia or dental abnormalities were not indicated [15].

Because of incomplete penetrance and variable phenotypic expression in VWS, the phenotype can mimic nsOFC. A family with VWS may exhibit barely visible lip pits/anomalies, dental abnormalities, or even no phenotypic abnormalities. Families are usually recruited for genetic studies based on the phenotype of the proband, so VWS may be overlooked if the proband does not display typical signs of VWS. This was demonstrated in a study by Leslie et al., when an a posteriori review of cases with suspected nsOFCs and deleterious *IRF6* variants revealed lip pits in many of the families [13]. Individuals with VWS2 (causal variants in *GRHL3*) are more likely to have CP and less likely to have CL/P and lip pits compared to individuals with VWS1 (causal variants in *IRF6*) [10], making the VWS2 phenotype even more similar to nsOFC. Furthermore, although nsOFCs are traditionally described as isolated anomalies without the presence of other malformations, patients with nsOFCs often have subphenotypes, such as dental anomalies [53], suggesting that the distinction between syOFCs and nsOFCs is imprecise. Based on this, it is questionable whether individuals with isolated clefts and *IRF6* or *GRHL3* variants really have nsOFCs. Nevertheless, Mangold et al. [15] have shown that deleterious *GRHL3* variants are more common in families with multiple CP cases, even if non-syndromic, and are inherited in an autosomal dominant manner, a fact not to be overlooked in genetic counselling. Individuals with non-syndromic CP and a *GRHL3* variant have a higher recurrence risk for CP with possible VWS signs in their offspring.

The following gene of interest, *TBX22*, has 9 exons, 8 of which are coding (exons 2–9) [46], and they encode a transcription factor with conserved T-box DNA-binding domain [16]. According to HGMD, missense, nonsense, splicing, and regulatory variants as well as small indels have been associated with CPX. Due to the location of *TBX22* on the X-chromosome, deleterious variants lead to a complete loss of function in males [16], which was also demonstrated in functional studies [54,55]. Although loss-of-function variants show high penetrance in males (CP in 96% and ankyloglossia in 79% of cases), haploinsufficient females usually show a milder phenotype (ankyloglossia only or no phenotype) [19]. In this study, we present a family (F-7) with a history of CP in males suggestive of an X-linked mode of inheritance, but the pattern did not match completely because the proband’s great-grandfather had bifid uvula and two sons with CP. We would like to emphasize the importance of using WES as a diagnostic tool, as without performing WES, we would not be able to detect CNVs in this family, so the deletion of *TBX22* would be missed. After analysing the data from WES and identifying the loss of all coding exons of *TBX22* in the proband with complete CP, we re-evaluated the family history and found ankyloglossia in individuals of all generations of the family, including putatively unaffected females. We validated the *TBX22* deletion by qPCR and confirmed the variant in two males (proband and one of his affected cousins) and their mothers with ankyloglossia and without CP. Samples from other family members were not available for the analysis. The phenotype of the family corresponds to the diagnosis of CPX. There is no evidence of a consanguine marriage between proband’s great-grandparents, possibly making the great-grandfather’s phenotype the result of different genetic or environmental factors. Interestingly, the great-grandfather also had ankyloglossia, a characteristic of CPX, indicating that there is also the probability that paternal heterodisomy of sex chromosomes occurred in his sons [56,57]. Marçano et al. [19] similarly identified a missense variant in *TBX22* in a family in which both the proband and his father had CP, but later, ankyloglossia was found in the proband’s mother and his maternal uncle, indicating that CPX was inherited from the mother and not from the father.

To complement the above findings, we examined WES data for 72 additional genes in the families without disease-causing variants in *IRF6*, *GRHL3*, or *TBX22* or without the diagnosis of VWS or CPX. We identified five rare variants, whose involvement in nsOFCs could not be clearly determined based on the available data, and nine rare variants that were excluded after the co-segregation analysis. Reporting these variants is important because it provides other researchers or clinicians with the knowledge that the specific variant has already been identified in an OFC case and helps them to include or exclude that variant as potentially causative in their cases. It also improves the classification of variants according to ACMG standards. The reason for being unsuccessful in finding any disease-causing variants in nsOFC cases might lie in our study design. Although we included all available Slovenian multi-case families, the number of families studied is small. Moreover, we screened a relatively small gene panel. Genes were selected through a systematic review of the genetic markers obtained from population case–control studies of nsOFCs [50]. Although we focused on screening genes implicated in nsOFCs in populations of European ancestry, some other studies have successfully screened nsOFC families using a broader range of candidate genes (more than 500) implicated in each form of OFCs (syOFCs and nsOFCs) and ethnicity [31,34]. This suggests that we may be successful in identifying monogenic causes in Slovenian nsOFC families if we expand the gene panel. In addition, selected genes were obtained from association studies examining disease-causing variants with relatively high population frequencies. The present study focused only on monogenic causes of nsOFCs, despite the fact that nsOFCs are commonly considered multifactorial disorders. We sought to reduce the impact of interactions between genetic and environmental factors in our cohort by including only families with multiple affected cases. Nevertheless, there is a likelihood that selected genes are involved in the complex aetiology in these families through the polygenic inheritance of variants with higher population frequencies.

## 4. Materials and Methods

### 4.1. Subject Recruitment

We recruited families with multiple cases of apparent nsOFCs (phenotypes resembling nsOFCs), that is, OFC families without or with additional minor facial clinical signs. In some families, additional facial signs were present in only some members. Our cohort mainly included multi-case families with nsOFCs but also multi-case families with suspected VWS and PRS. Exclusion criteria included single-case families, families where the subjects had OFC in combination with defects of other organ systems (e.g., congenital heart defects), or with previously confirmed chromosomal abnormalities.

The majority of the probands and their affected and non-affected family members were recruited from September 2019 to February 2021 at the Department of Maxillofacial and Oral Surgery, University Medical Centre Ljubljana in Ljubljana, Slovenia. The probands’ mothers were asked to fill in the questionnaire in order to determine the family history and evaluate their medical conditions or exposure to environmental risk factors during pregnancy. The diagnosis of OFC was based on a thorough clinical examination and assessment of the diagnostic data from medical records by a maxillofacial surgeon (A.E.).

Overall, we included 34 families with two or more members affected with apparent nsOFCs, where 24 families had members with nsOFCs, three families had at least one member with signs of PRS, and seven families had at least one member with lip pits, suggesting the diagnosis of VWS. As all the cases of OFCs in Slovenia are treated in one tertiary centre (Department of Maxillofacial and Oral Surgery, University Medical Centre Ljubljana), we included all of the available multi-case families from Slovenia. All probands and their family members were of European descent.

Altogether, the initial analysis included the selection of 39 affected subjects (22 males, 17 females) drawn from 34 families; one affected subject in the case of 29 families, and two affected siblings/cousins in the case of five families. Apart from lip pits, seven subjects from seven families with presumably VWS had bilateral CLP (*n* = 1), unilateral CLP (*n* = 2), complete CP (*n* = 3), or soft CP (*n* = 1). The remaining 32 subjects from 27 families had bilateral CL/P (*n* = 6), unilateral CL/P (*n* = 13), complete CP (*n* = 7), soft CP (*n* = 3), or PRS (*n* = 3). We recruited between 1 and 7 affected and non-affected family members per multi-case family, depending on their family history and willingness to cooperate.

All the subjects or their parents/legal guardians (for subjects under 15 years) signed the informed consent form. The study protocols were approved by the National Medical Ethics Committee of the Republic of Slovenia (0120-211/2019/3).

### 4.2. DNA Extraction and Genetic Testing

EDTA blood (venous/capillary) samples or buccal swab samples were collected, and genomic DNA was extracted using three different commercial kits: FlexiGene DNA kit (Qiagen, Hilden, Germany), QIAamp DNA Mini kit (Qiagen, Hilden, Germany), or MasterPure complete DNA and RNA purification kit (Epicentre (Illumina), Madison, WI, USA), according to the manufacturers’ instructions.

The Multiplex ligation-dependent probe amplification (MLPA) assay was performed on samples of all the probands using the SALSA MLPA Probemix P245-B1 Microdeletion Syndromes-1A (MRC-Holland, Amsterdam, The Netherlands), according to the manufacturer’s instructions. The kit tested for the presence of deletions/duplications in various chromosomal regions involved in selected microdeletion and microduplication syndromes, including the 22q11.2 region, but no aberrations were detected.

#### 4.2.1. Two-Step Sequence Analysis

The first step of the sequence analysis comprised screening the probands for disease-causing variants in the three genes known to be implicated in VWS and CPX: *IRF6*, *GRHL3*, and *TBX22*.

In seven affected subjects from six families, Sanger sequencing was used to analyse the three genes due to the lack of high-quality DNA. First, protein-coding exons and flanking intronic regions were amplified by PCR using a HOT FIREPol^®^ DNA Polymerase kit (Solis BioDyne, Tartu, Estonia) and in-house primer pairs designed using Primer3 (v4.1.0) software (Table S4) [58]. The PCRs were performed according to the manufacturer’s instructions. The PCR products and primers were subsequently sent to McLab (San Francisco, CA, USA) for Sanger sequencing. Sufficient high-quality DNA was available for the remainder of the affected subjects (*n* = 32), so *IRF6, GRHL3*, and *TBX22* were analysed in these samples by WES.

In the second step of the sequence analysis, the WES data of the subjects lacking disease-causing variants in the three selected genes were further filtered for variants in 72 additional genes (Table S5). The selection criteria for nominating candidate genes were based on information from an extensive systematic review in which we compiled data from 84 population-based case–control studies and investigated genetic risk factors for nsOFCs in populations of European ancestry. A meta-analysis was performed for repeatedly reported genetic variants from 43 of these studies. The genetic variants from 84 studies that were not included in the meta-analysis were only reviewed [50]. We selected all genes that were included in the meta-analysis (statistically significant and not significant) because these genes were most frequently studied in populations of European ancestry. Candidate genes were also selected based on variants that were not included in the meta-analysis but were significantly associated with nsOFCs in one of the 84 studies. A few studies investigated rare variants by sequencing the coding regions of specific genes. Because it is more difficult to demonstrate a statistically significant association with the abnormality for rare variants, we also included genes that were studied in this way. On the other hand, we did not consider genetic variants located in non-coding regions or variants for which the corresponding gene was not mentioned in the studies.

#### 4.2.2. Whole Exome Sequencing and Data Analysis

WES was carried out at the CeGaT GmbH (Tübingen, Germany) using the Twist Human Core Exome Plus Kit (Twist Bioscience, San Francisco, CA, USA). The paired-end sequencing (2 × 100 bp reads) was performed on a NovaSeq 6000 (Illumina, San Diego, CA, USA). After sequencing, reads were demultiplexed (Illumina bcl2fastq 2.20) and adapters were trimmed (Skewer 0.2.2) [59]. The generated reads were aligned to the human reference genome (hg19-cegat) using a Burrows-Wheeler Aligner (BWA-mem version 0.7.17-cegat) [60]. Reads at the target regions were locally realigned using ABRA (version 2.18) to improve indel detection [61]. A CeGaT proprietary tool was used to discard duplicated reads and reads that aligned with identical mapping scores to more than one locus. The achieved average coverage was >107x. The variants were detected and annotated using an additional CeGaT proprietary software. The annotation was performed using various public databases (Ensembl (v100) [46], RefSeq Curated (20200723) [62], CCDS (r22) [63], GnomAD (2.1.1 (exonic), 3.1 (genomic)) [52], dbSNP154 [64], Gencode 34 [65]). The CNVs were also computed using CeGaT internally developed method. The method compares the expected number of reads on the target loci (coverage in a number of CeGaT reference samples) with the observed number (coverage in the tested samples) [66].

The resulting variants were analysed in the affected subjects of each family independently. We have only considered rare variants with a MAF ≤0.01 in gnomAD and/or dbSNP154, of which we have only examined LoF variants (e.g., nonsense, nonstop, initiation codon, essential/canonical splice site variants, frameshift indels, and single-exon or multi-exon deletions), microduplications, splice-region variants, missense single nucleotide variants (SNVs), and in-frame indels. Other variants were discarded. Tools provided by The Ensembl Variant Effect Predictor (VEP) [67] and Franklin (Genoox) [68] platform were used to predict the consequences of each variant in silico. Missense SNVs were suspected to be protein-altering if predicted to be deleterious/damaging by at least three in silico deleteriousness/conservation prediction tools (SIFT [69], PolyPhen-2 [70], MutationAssessor [71], MutationTaster [72], FATHMM [73], CADD [74], MetaLR [75], REVEL [76] or GERP++ [77]) or predicted “deleterious”/“uncertain” by Franklin’s aggregated prediction. The potential of splice-region SNVs to alter splicing was predicted by using splice site prediction tools (MaxEntScan [78], dbscSNV Ada [79], and SpliceAI [80]), MutationTester, and CADD. All the variants that met the inclusion criteria were visually inspected by Integrative Genomics Viewer (IGV) [81] to confirm them as “true” variants.

Franklin (Genoox) platform [68] was also used to classify variants based on the ACMG guidelines [82]. In accordance with these criteria, the variants were classified into five groups as benign, likely benign, variant of uncertain significance (VUS), likely pathogenic, and pathogenic. Our analysis focused only on variants that were classified as VUS, likely pathogenic, and pathogenic [82]. Lastly, we reviewed the literature, HGMD Professional 2022.2 (Qiagen, Hilden, Germany), ClinVar database [83], and DECIPHER v11.12 [84] to identify known disease-causing variants.

#### 4.2.3. Variant Validation and Co-Segregation Analysis

All the putative variants found in the probands were validated, and co-segregation analysis was also performed on their available affected and non-affected family members. The SNVs/indels and CNVs were confirmed using Sanger sequencing and qPCR, respectively. To further confirm the presence of CNVs and more precisely determine their location and size, we also performed array CGH on the proband.

DNA sequences with the SNVs or indels were amplified by PCR using HOT FIREPol^®^ DNA Polymerase kit (Solis BioDyne, Tartu, Estonia) and in-house primer pairs designed using Primer3 (v4.1.0) (Table S6). The reactions were performed according to the manufacturer’s instructions. PCR products and primers were later sent to McLab (San Francisco, USA) for Sanger sequencing.

The qPCR was used to confirm CNVs, i.e., the deletion of all the coding exons of *TBX22*. We modified the method described by Weksberg et al. [85]. Reactions were performed with HOT FIREPol^®^ EvaGreen^®^ qPCR Supermix (Solis BioDyne, Tartu, Estonia) and in-house primer pairs designed using Primer3 (v4.1.0). Designed primer pairs targeted eight coding exons of *TBX22* (exons 2–9) and two exons of the two selected reference genes, *G6PD* (exon 3) and *IRF6* (exon 5). We chose *G6PD* because it is a commonly used X-linked housekeeping gene [85] and *IRF6* because primer pair was available. Primer-BLAST (NCBI) [86] was used to ensure the primers were specific for the target sequences. We optimized the concentration and annealing temperature for each primer pair, which are listed in Table S7 along with the genomic targets, amplicon sizes, and optimized conditions. The qPCR was performed according to the manufacturers’ instructions using the LightCycler^®^ 480 Real-Time PCR System (Roche, Basel, Switzerland), and the resulting data were analysed with LightCycler^®^ 480 software release 1.5.1.62 SP3 (Roche, Basel, Switzerland). Melting curve analysis was performed to confirm the specificity of each amplification. Due to the location of *TBX22* on the X chromosome and the associated difference in allele numbers between the sexes, male (n = 3) and female (n = 4) genomic control DNA samples were included in the analysis. In addition, two separate standard curves were generated for all qPCR reactions using twofold dilution series of a male and a female control DNA sample. Reactions were performed in triplicate and PCR-grade water was used as a blank.

Instructions by Weksberg et al. [85] were followed for data analysis and calculation of the fold change in copy number (∆KCt) for each sample. The average Ct values of the target region (*TBX22* exons) for each control and test sample were normalized using the average Ct values of the reference gene (*G6PD* or *IRF6*) and slope values derived from standard curves. To control for variability between sexes as a result of different allele numbers, we employed the equation of Weksberg et al. [85] for male and female (control and test) samples separately. The fold change in copy number (∆KCt; copy number of each *TBX22* exon) was then determined by comparing the normalized data of the control and test samples (male–male and female–female). ∆KCt values of 0 ± 0.35 indicate no copy number change or no genetic abnormality (in males and females), whereas −1 ± 0.35 indicates a loss of one allelic copy (the deletion of the *TBX22* exon) in females, who normally carry two copies. In the male samples, the loss of a single allelic copy of each *TBX22* exon was detected when no qPCR product was present or the Ct value was similar to the blank Ct value (i.e., no peak was generated in the melting curve analysis). The quality of the DNA from these samples was verified by the presence of a qPCR product when reference genes were amplified.

In addition, array CGH was performed on the sample from the proband with the *TBX22* deletion to localise the identified CNV and its size. Array CGH analysis was performed using a commercial oligonucleotide array (Agilent 180K Baylor Oligo, Agilent Technologies, Santa Clara, CA, USA) and a sex-matched human reference DNA sample (Agilent Technologies, Santa Clara, CA, USA). Data were analysed using Cytogenomics 5.1.2.1 Software (Agilent Technologies, Santa Clara, CA, USA).

## 5. Conclusions

The present comprehensive genetic study is the first study investigating Slovenian families with multiple cases of OFCs. Its main outcome is the identification of novel genetic variants in known OFC genes and their potential application as a diagnostic approach to distinguish between nsOFCs and syOFCs. The sequencing of known OFC genes is clearly a powerful tool to make or improve a diagnosis. We recruited families with apparent nsOFCs (i.e., OFC families without or with additional minor facial signs). Using WES and Sanger sequencing, we screened the selected 75 genes and identified six disease-causing variants in 7 of 34 families (20.6%). These variants were located in 3 genes, *IRF6*, *GRHL3*, and *TBX22*. With the identification of four disease-causing SNVs in *IRF6*, one of which was novel, we confirmed the VWS1 diagnosis in five families with OFC and lip pits. Interestingly, we also identified two syndromic forms of OFCs in our cohort of suspected nsOFCs. A novel splice-altering SNV in *GRHL3* identified a family with VWS2, and the novel CNV, the deletion of *TBX22* coding exons, revealed a familial CPX. Although we also identified and analysed many rare variants in probands with nsOFCs, the involvement of nine SNVs/indels was excluded after co-segregation analysis, whereas the results for five SNVs/indels are inconclusive. Our sequencing approach and gene selection were successful in identifying syOFC families with monogenic inheritance patterns in a cohort of apparent nsOFCs, suggesting that WES is useful for diagnostic purposes in OFC families with minor additional clinical signs and multiple cases. Our results show that the sequencing of *IRF6*, *GRHL3*, and *TBX22* has a high diagnostic yield. This is particularly important in cases where the phenotype is complex and difficult to characterize clinically. However, our approach was unsuccessful in identifying the monogenic cause of nsOFCs. Additional approaches that consider multifactorial aetiology should be used to identify the complete genetic aetiology of nsOFCs.

## Figures and Tables

**Table 1:** *IRF6* and *GRHL3* variants that confirm the diagnosis of Van der Woude syndrome in six families.

	Genomic Location (hg19)	Exon	Ref>Alt	Variant Name ^a^	Amino Acid Change ^b^	Variant Type	ACMG Class	Published Reference
***IRF6***								
F-1	Chr1:209,974,625	3	C>T	c.134G>A rs121434229	p.Arg45Gln	missense	likely pathogenic	[37]
F-2	Chr1:209,965,659	6	G>A	c.622C>T	p.Gln208*	nonsense	pathogenic	[38]
F-3	Chr1:209,964,212	7	AC>A	c.687delG	p.Lys229Asnfs*13	frameshift	likely pathogenic	novel variant
F-4, F-5	Chr1:209,961,935	9	G>A	c.1234C>T rs1553247595	p.Arg412*	nonsense	pathogenic	[4,9,38,39,40,41,42,43]
***GRHL3***								
F-6	Chr1:24,669,262	10	G>T	c.1285G>T	p.Gly429Cys	splice-site	VUS	novel variant

F-no., the family identifier; Ref>Alt, reference and alternative allele, where reference allele is listed first. ^a^ The position of the nucleotide change, determined based on the canonical transcript of *IRF6* (NM_006147.4) or *GRHL3* (NM_198173.3), and ‘rsID’ number if available. ^b^ The position of amino acid change, determined based on the canonical protein IRF6 (NP_006138.1) or GRHL3 (NP_937816.1).

## Data Availability

The data presented in this study are available on request from the corresponding author. The data are not publicly available due to privacy and ethical restrictions.

## Supplementary Materials

The following supporting information can be downloaded at: https://www.mdpi.com/article/10.3390/ijms24054262/s1.

## References

[1] Gundlach K.K.H., Maus C. (2006). Epidemiological Studies on the Frequency of Clefts in Europe and World-Wide. J. Cranio-Maxillofac. Surg..

[2] Koželj V. (2015). Računalniški informacijski sistem za spremljanje orofacialnih shiz v Sloveniji kot vir za epidemiološko analizo obdobij 1873–1993 in 1993–2012. Zobozdrav. Vestn..

[3] Fraser F.C. (1955). Thoughts on the Etiology of Clefts of the Palate and Lip. Acta Genet. Stat. Med..

[4] Kondo S., Schutte B.C., Richardson R.J., Bjork B.C., Knight A.S., Watanabe Y., Howard E., Ferreira de Lima R.L.L., Daack-Hirsch S., Sander A. (2002). Mutations in IRF6 Cause Van Der Woude and Popliteal Pterygium Syndromes. Nat. Genet..

[5] Schutte B.C., Murray J.C. (1999). The Many Faces and Factors of Orofacial Clefts. Hum. Mol. Genet..

[6] Rittler M., Cosentino V., López-Camelo J.S., Murray J.C., Wehby G., Castilla E.E. (2011). Associated Anomalies among Infants with Oral Clefts at Birth and during a 1-Year Follow-Up. Am. J. Med. Genet. A.

[7] Venkatesh R. (2009). Syndromes and Anomalies Associated with Cleft. Indian J. Plast. Surg..

[8] Gorlin R.J., Cohen M.M., Hennekam R.C. (2001). Syndromes of the Head and Neck.

[9] de Lima R.L.L.F., Hoper S.A., Ghassibe M., Cooper M.E., Rorick N.K., Kondo S., Katz L., Marazita M.L., Compton J., Bale S. (2009). Prevalence and Nonrandom Distribution of Exonic Mutations in Interferon Regulatory Factor 6 in 307 Families with Van Der Woude Syndrome and 37 Families with Popliteal Pterygium Syndrome. Genet. Med..

[10] Peyrard-Janvid M., Leslie E.J., Kousa Y.A., Smith T.L., Dunnwald M., Magnusson M., Lentz B.A., Unneberg P., Fransson I., Koillinen H.K. (2014). Dominant Mutations in GRHL3 Cause Van Der Woude Syndrome and Disrupt Oral Periderm Development. Am. J. Hum. Genet..

[11] Burdick A.B., Bixler D., Puckett C.L. (1985). Genetic Analysis in Families with van Der Woude Syndrome. J. Craniofac. Genet. Dev. Biol..

[12] Lacombe D., Pedespan J.M., Fontan D., Chateil J.F., Verloes A. (1995). Phenotypic Variability in van Der Woude Syndrome. Genet. Couns. Geneva Switz..

[13] Leslie E.J., Koboldt D.C., Kang C.J., Ma L., Hecht J.T., Wehby G.L., Christensen K., Czeizel A.E., Deleyiannis F.W.-B., Fulton R.S. (2016). IRF6 Mutation Screening in Non-Syndromic Orofacial Clefting: Analysis of 1521 Families. Clin. Genet..

[14] Zucchero T.M., Cooper M.E., Maher B.S., Daack-Hirsch S., Nepomuceno B., Ribeiro L., Caprau D., Christensen K., Suzuki Y., Machida J. (2004). Interferon Regulatory Factor 6 (IRF6) Gene Variants and the Risk of Isolated Cleft Lip or Palate. N. Engl. J. Med..

[15] Mangold E., Böhmer A.C., Ishorst N., Hoebel A.-K., Gültepe P., Schuenke H., Klamt J., Hofmann A., Gölz L., Raff R. (2016). Sequencing the GRHL3 Coding Region Reveals Rare Truncating Mutations and a Common Susceptibility Variant for Nonsyndromic Cleft Palate. Am. J. Hum. Genet..

[16] Braybrook C., Doudney K., Marçano A.C., Arnason A., Bjornsson A., Patton M.A., Goodfellow P.J., Moore G.E., Stanier P. (2001). The T-Box Transcription Factor Gene TBX22 Is Mutated in X-Linked Cleft Palate and Ankyloglossia. Nat. Genet..

[17] Lowry R.B. (1970). Sex-Linked Cleft Palate in a British Columbia Indian Family. Pediatrics.

[18] Björnsson A., Arnason A., Tippet P. (1989). X-Linked Cleft Palate and Ankyloglossia in an Icelandic Family. Cleft Palate J..

[19] Marçano A.C.B., Doudney K., Braybrook C., Squires R., Patton M.A., Lees M.M., Richieri-Costa A., Lidral A.C., Murray J.C., Moore G.E. (2004). TBX22 Mutations Are a Frequent Cause of Cleft Palate. J. Med. Genet..

[20] Kot M., Kruk-Jeromini J. (2007). Analysis of Family Incidence of Cleft Lip and/or Palate. Med. Sci. Monit. Int. Med. J. Exp. Clin. Res..

[21] Grosen D., Chevrier C., Skytthe A., Bille C., Molsted K., Sivertsen A., Murray J.C., Christensen K. (2010). A Cohort Study of Recurrence Patterns among More than 54 000 Relatives of Oral Cleft Cases in Denmark: Support for the Multifactorial Threshold Model of Inheritance. J. Med. Genet..

[22] Little J., Bryan E. (1986). Congenital Anomalies in Twins. Semin. Perinatol..

[23] Grosen D., Bille C., Petersen I., Skytthe A., von Bornemann Hjelmborg J., Pedersen J.K., Murray J.C., Christensen K. (2011). Risk of Oral Clefts in Twins. Epidemiology.

[24] Rahimov F., Jugessur A., Murray J.C. (2012). Genetics of Nonsyndromic Orofacial Clefts. Cleft Palate-Craniofacial J. Off. Publ. Am. Cleft Palate-Craniofacial Assoc..

[25] Peng H.-H., Chang N.-C., Chen K.-T., Lu J.-J., Chang P.-Y., Chang S.-C., Wu-Chou Y.-H., Chou Y.-T., Phang W., Cheng P.-J. (2016). Nonsynonymous Variants in MYH9 and ABCA4 Are the Most Frequent Risk Loci Associated with Nonsyndromic Orofacial Cleft in Taiwanese Population. BMC Med. Genet..

[26] Shibano M., Watanabe A., Takano N., Mishima H., Kinoshita A., Yoshiura K.-I., Shibahara T. (2020). Target Capture/Next-Generation Sequencing for Nonsyndromic Cleft Lip and Palate in the Japanese Population. Cleft Palate-Craniofacial J. Off. Publ. Am. Cleft Palate-Craniofacial Assoc..

[27] Reich D.E., Lander E.S. (2001). On the Allelic Spectrum of Human Disease. Trends Genet..

[28] Ludwig K.U., Böhmer A.C., Bowes J., Nikolic M., Ishorst N., Wyatt N., Hammond N.L., Gölz L., Thieme F., Barth S. (2017). Imputation of Orofacial Clefting Data Identifies Novel Risk Loci and Sheds Light on the Genetic Background of Cleft Lip ± Cleft Palate and Cleft Palate Only. Hum. Mol. Genet..

[29] Manolio T.A., Collins F.S., Cox N.J., Goldstein D.B., Hindorff L.A., Hunter D.J., McCarthy M.I., Ramos E.M., Cardon L.R., Chakravarti A. (2009). Finding the Missing Heritability of Complex Diseases. Nature.

[30] Bureau A., Parker M.M., Ruczinski I., Taub M.A., Marazita M.L., Murray J.C., Mangold E., Noethen M.M., Ludwig K.U., Hetmanski J.B. (2014). Whole Exome Sequencing of Distant Relatives in Multiplex Families Implicates Rare Variants in Candidate Genes for Oral Clefts. Genetics.

[31] Pengelly R.J., Arias L., Martínez J., Upstill-Goddard R., Seaby E.G., Gibson J., Ennis S., Collins A., Briceño I. (2016). Deleterious Coding Variants in Multi-Case Families with Non-Syndromic Cleft Lip and/or Palate Phenotypes. Sci. Rep..

[32] Liu H., Busch T., Eliason S., Anand D., Bullard S., Gowans L.J.J., Nidey N., Petrin A., Augustine-Akpan E.-A., Saadi I. (2017). Exome Sequencing Provides Additional Evidence for the Involvement of ARHGAP29 in Mendelian Orofacial Clefting and Extends the Phenotypic Spectrum to Isolated Cleft Palate. Birth Defects Res..

[33] Hoebel A.K., Drichel D., van de Vorst M., Böhmer A.C., Sivalingam S., Ishorst N., Klamt J., Gölz L., Alblas M., Maaser A. (2017). Candidate Genes for Nonsyndromic Cleft Palate Detected by Exome Sequencing. J. Dent. Res..

[34] Basha M., Demeer B., Revencu N., Helaers R., Theys S., Bou Saba S., Boute O., Devauchelle B., Francois G., Bayet B. (2018). Whole Exome Sequencing Identifies Mutations in 10% of Patients with Familial Non-Syndromic Cleft Lip and/or Palate in Genes Mutated in Well-Known Syndromes. J. Med. Genet..

[35] Demeer B., Revencu N., Helaers R., Devauchelle B., François G., Bayet B., Vikkula M. (2018). Unmasking Familial CPX by WES and Identification of Novel Clinical Signs. Am. J. Med. Genet. A.

[36] Cox L.L., Cox T.C., Moreno Uribe L.M., Zhu Y., Richter C.T., Nidey N., Standley J.M., Deng M., Blue E., Chong J.X. (2018). Mutations in the Epithelial Cadherin-P120-Catenin Complex Cause Mendelian Non-Syndromic Cleft Lip with or without Cleft Palate. Am. J. Hum. Genet..

[37] Kayano S., Kure S., Suzuki Y., Kanno K., Aoki Y., Kondo S., Schutte B.C., Murray J.C., Yamada A., Matsubara Y. (2003). Novel IRF6 Mutations in Japanese Patients with Van Der Woude Syndrome: Two Missense Mutations (R45Q and P396S) and a 17-Kb Deletion. J. Hum. Genet..

[38] Birkeland A.C., Larrabee Y., Kent D.T., Flores C., Su G.H., Lee J.H., Haddad J. (2011). Novel IRF6 Mutations in Honduran Van Der Woude Syndrome Patients. Mol. Med. Rep..

[39] Li S., Zhang X., Chen D., Zhao W., Zhang X., Jiao J., Guo L., Yin L., Song X., Liang C. (2018). Association between Genotype and Phenotype of Virulence Gene in Van Der Woude Syndrome Families. Mol. Med. Rep..

[40] Peyrard-Janvid M., Pegelow M., Koillinen H., Larsson C., Fransson I., Rautio J., Hukki J., Larson O., Karsten A.L.-A., Kere J. (2005). Novel and de Novo Mutations of the IRF6 Gene Detected in Patients with Van Der Woude or Popliteal Pterygium Syndrome. Eur. J. Hum. Genet..

[41] Tan E.-C., Lim E.C.-P., Yap S.-H., Lee S.-T., Cheng J., Por Y.-C., Yeow V. (2008). Identification of IRF6 Gene Variants in Three Families with Van Der Woude Syndrome. Int. J. Mol. Med..

[42] Malik S., Kakar N., Hasnain S., Ahmad J., Wilcox E., Naz S. (2010). Epidemiology of Van Der Woude Syndrome from Mutational Analyses in Affected Patients from Pakistan. Clin. Genet..

[43] Du X., Tang W., Tian W., Li S., Li X., Liu L., Zheng X., Chen X., Lin Y., Tang Y. (2006). Novel *IRF6* Mutations in Chinese Patients with Van Der Woude Syndrome. J. Dent. Res..

[44] Kwa M.Q., Huynh J., Reynolds E.C., Hamilton J.A., Scholz G.M. (2015). Disease-Associated Mutations in IRF6 and RIPK4 Dysregulate Their Signalling Functions. Cell. Signal..

[45] Desmyter L., Ghassibe M., Revencu N., Boute O., Lees M., François G., Verellen-Dumoulin C., Sznajer Y., Moncla A., Benateau H. (2010). *IRF6* Screening of Syndromic and a Priori Non-Syndromic Cleft Lip and Palate Patients: Identification of a New Type of Minor VWS Sign. Mol. Syndromol..

[46] Yates A.D., Achuthan P., Akanni W., Allen J., Allen J., Alvarez-Jarreta J., Amode M.R., Armean I.M., Azov A.G., Bennett R. (2019). Ensembl 2020. Nucleic Acids Res..

[47] Eroshkin A., Mushegian A. (1999). Conserved Transactivation Domain Shared by Interferon Regulatory Factors and Smad Morphogens. J. Mol. Med. Berl. Ger..

[48] Leslie E.J., Standley J., Compton J., Bale S., Schutte B.C., Murray J.C. (2013). Comparative Analysis of IRF6 Variants in Families with Van Der Woude Syndrome and Popliteal Pterygium Syndrome Using Public Whole-Exome Databases. Genet. Med. Off. J. Am. Coll. Med. Genet..

[49] Cooper D.N., Youssoufian H. (1988). The CpG Dinucleotide and Human Genetic Disease. Hum. Genet..

[50] Slavec L., Karas Kuželički N., Locatelli I., Geršak K. (2022). Genetic Markers for Non-Syndromic Orofacial Clefts in Populations of European Ancestry: A Meta-Analysis. Sci. Rep..

[51] Eshete M.A., Liu H., Li M., Adeyemo W.L., Gowans L.J.J., Mossey P.A., Busch T., Deressa W., Donkor P., Olaitan P.B. (2018). Loss-of-Function GRHL3 Variants Detected in African Patients with Isolated Cleft Palate. J. Dent. Res..

[52] Karczewski K.J., Francioli L.C., Tiao G., Cummings B.B., Alföldi J., Wang Q., Collins R.L., Laricchia K.M., Ganna A., Birnbaum D.P. (2020). The Mutational Constraint Spectrum Quantified from Variation in 141,456 Humans. Nature.

[53] Letra A., Menezes R., Granjeiro J.M., Vieira A.R. (2007). Defining Subphenotypes for Oral Clefts Based on Dental Development. J. Dent. Res..

[54] Andreou A.M., Pauws E., Jones M.C., Singh M.K., Bussen M., Doudney K., Moore G.E., Kispert A., Brosens J.J., Stanier P. (2007). TBX22 Missense Mutations Found in Patients with X-Linked Cleft Palate Affect DNA Binding, Sumoylation, and Transcriptional Repression. Am. J. Hum. Genet..

[55] Pauws E., Hoshino A., Bentley L., Prajapati S., Keller C., Hammond P., Martinez-Barbera J.-P., Moore G.E., Stanier P. (2009). Tbx22 Null Mice Have a Submucous Cleft Palate Due to Reduced Palatal Bone Formation and Also Display Ankyloglossia and Choanal Atresia Phenotypes. Hum. Mol. Genet..

[56] Ferrier R.A., Lowry R.B., Lemire E.G., Stoeber G.P., Howard J., Parboosingh J.S. (2009). Father-to-Son Transmission of an X-Linked Gene: A Case of Paternal Sex Chromosome Heterodisomy. Am. J. Med. Genet. A.

[57] del Gaudio D., Shinawi M., Astbury C., Tayeh M.K., Deak K.L., Raca G. (2020). Diagnostic Testing for Uniparental Disomy: A Points to Consider Statement from the American College of Medical Genetics and Genomics (ACMG). Genet. Med..

[58] Untergasser A., Cutcutache I., Koressaar T., Ye J., Faircloth B.C., Remm M., Rozen S.G. (2012). Primer3—New Capabilities and Interfaces. Nucleic Acids Res..

[59] Jiang H., Lei R., Ding S.-W., Zhu S. (2014). Skewer: A Fast and Accurate Adapter Trimmer for next-Generation Sequencing Paired-End Reads. BMC Bioinform..

[60] Li H., Durbin R. (2009). Fast and Accurate Short Read Alignment with Burrows-Wheeler Transform. Bioinformatics.

[61] Mose L.E., Wilkerson M.D., Hayes D.N., Perou C.M., Parker J.S. (2014). ABRA: Improved Coding Indel Detection via Assembly-Based Realignment. Bioinformatics.

[62] O’Leary N.A., Wright M.W., Brister J.R., Ciufo S., Haddad D., McVeigh R., Rajput B., Robbertse B., Smith-White B., Ako-Adjei D. (2016). Reference Sequence (RefSeq) Database at NCBI: Current Status, Taxonomic Expansion, and Functional Annotation. Nucleic Acids Res..

[63] Pujar S., O’Leary N.A., Farrell C.M., Loveland J.E., Mudge J.M., Wallin C., Girón C.G., Diekhans M., Barnes I., Bennett R. (2018). Consensus Coding Sequence (CCDS) Database: A Standardized Set of Human and Mouse Protein-Coding Regions Supported by Expert Curation. Nucleic Acids Res..

[64] Sherry S.T., Ward M.H., Kholodov M., Baker J., Phan L., Smigielski E.M., Sirotkin K. (2001). DbSNP: The NCBI Database of Genetic Variation. Nucleic Acids Res..

[65] Frankish A., Diekhans M., Jungreis I., Lagarde J., Loveland J.E., Mudge J.M., Sisu C., Wright J.C., Armstrong J., Barnes I. (2021). GENCODE 2021. Nucleic Acids Res..

[66] Amaral T., Schulze M., Sinnberg T., Nieser M., Martus P., Battke F., Garbe C., Biskup S., Forschner A. (2020). Are Pathogenic Germline Variants in Metastatic Melanoma Associated with Resistance to Combined Immunotherapy?. Cancers.

[67] McLaren W., Gil L., Hunt S.E., Riat H.S., Ritchie G.R.S., Thormann A., Flicek P., Cunningham F. (2016). The Ensembl Variant Effect Predictor. Genome Biol..

[68] Franklin by Genoox. https://franklin.genoox.com.

[69] Sim N.-L., Kumar P., Hu J., Henikoff S., Schneider G., Ng P.C. (2012). SIFT Web Server: Predicting Effects of Amino Acid Substitutions on Proteins. Nucleic Acids Res..

[70] Adzhubei I.A., Schmidt S., Peshkin L., Ramensky V.E., Gerasimova A., Bork P., Kondrashov A.S., Sunyaev S.R. (2010). A Method and Server for Predicting Damaging Missense Mutations. Nat. Methods.

[71] Reva B., Antipin Y., Sander C. (2007). Determinants of Protein Function Revealed by Combinatorial Entropy Optimization. Genome Biol..

[72] Schwarz J.M., Cooper D.N., Schuelke M., Seelow D. (2014). MutationTaster2: Mutation Prediction for the Deep-Sequencing Age. Nat. Methods.

[73] Shihab H.A., Gough J., Mort M., Cooper D.N., Day I.N., Gaunt T.R. (2014). Ranking Non-Synonymous Single Nucleotide Polymorphisms Based on Disease Concepts. Hum. Genom..

[74] Kircher M., Witten D.M., Jain P., O’Roak B.J., Cooper G.M., Shendure J. (2014). A General Framework for Estimating the Relative Pathogenicity of Human Genetic Variants. Nat. Genet..

[75] Dong C., Wei P., Jian X., Gibbs R., Boerwinkle E., Wang K., Liu X. (2015). Comparison and Integration of Deleteriousness Prediction Methods for Nonsynonymous SNVs in Whole Exome Sequencing Studies. Hum. Mol. Genet..

[76] Ioannidis N.M., Rothstein J.H., Pejaver V., Middha S., McDonnell S.K., Baheti S., Musolf A., Li Q., Holzinger E., Karyadi D. (2016). REVEL: An Ensemble Method for Predicting the Pathogenicity of Rare Missense Variants. Am. J. Hum. Genet..

[77] Davydov E.V., Goode D.L., Sirota M., Cooper G.M., Sidow A., Batzoglou S. (2010). Identifying a High Fraction of the Human Genome to Be under Selective Constraint Using GERP++. PLoS Comput. Biol..

[78] Yeo G., Burge C.B. (2004). Maximum Entropy Modeling of Short Sequence Motifs with Applications to RNA Splicing Signals. J. Comput. Biol..

[79] Jian X., Boerwinkle E., Liu X. (2014). In Silico Prediction of Splice-Altering Single Nucleotide Variants in the Human Genome. Nucleic Acids Res..

[80] Jaganathan K., Kyriazopoulou Panagiotopoulou S., McRae J.F., Darbandi S.F., Knowles D., Li Y.I., Kosmicki J.A., Arbelaez J., Cui W., Schwartz G.B. (2019). Predicting Splicing from Primary Sequence with Deep Learning. Cell.

[81] Robinson J.T., Thorvaldsdóttir H., Wenger A.M., Zehir A., Mesirov J.P. (2017). Variant Review with the Integrative Genomics Viewer. Cancer Res..

[82] Richards S., Aziz N., Bale S., Bick D., Das S., Gastier-Foster J., Grody W.W., Hegde M., Lyon E., Spector E. (2015). Standards and Guidelines for the Interpretation of Sequence Variants: A Joint Consensus Recommendation of the American College of Medical Genetics and Genomics and the Association for Molecular Pathology. Genet. Med..

[83] Landrum M.J., Lee J.M., Benson M., Brown G.R., Chao C., Chitipiralla S., Gu B., Hart J., Hoffman D., Jang W. (2018). ClinVar: Improving Access to Variant Interpretations and Supporting Evidence. Nucleic Acids Res..

[84] Firth H.V., Richards S.M., Bevan A.P., Clayton S., Corpas M., Rajan D., Vooren S.V., Moreau Y., Pettett R.M., Carter N.P. (2009). DECIPHER: Database of Chromosomal Imbalance and Phenotype in Humans Using Ensembl Resources. Am. J. Hum. Genet..

[85] Weksberg R., Hughes S., Moldovan L., Bassett A.S., Chow E.W., Squire J.A. (2005). A Method for Accurate Detection of Genomic Microdeletions Using Real-Time Quantitative PCR. BMC Genom..

[86] Ye J., Coulouris G., Zaretskaya I., Cutcutache I., Rozen S., Madden T.L. (2012). Primer-BLAST: A Tool to Design Target-Specific Primers for Polymerase Chain Reaction. BMC Bioinform..

