# Supplementary material for: A Comprehensive Genetic Analysis of Slovenian Families with Multiple Cases of Orofacial Clefts Reveals Novel Variants in the Genes IRF6, GRHL3, and TBX22
Source: Int J Mol Sci. 2023 Feb 21;24(5):4262. doi: 10.3390/ijms24054262 (PMC10002089; doi:10.3390/ijms24054262)
Supplement: Supplementary file 1 [file ijms-24-04262-s001.zip › ijms-2089997-supplementary.pdf]

Supplementary Materials

Tables S1–S7; Figures S1 and S2

A Comprehensive Genetic Analysis of Slovenian Families with Multiple Cases of Orofacial Clefts Reveals Novel Variants in the Genes *IRF6*, *GRHL3*, and *TBX22*

Lara Slavec[1,2], Ksenija Geršak[1,3], Andreja Eberlinc[4], Tinka Hovnik[5,6], Luca Lovrečić[3,7], Irena Mlinarič-Raščan[2], Nataša Karas Kuželički[2]

[1] University Medical Centre Ljubljana, Division of Gynaecology and Obstetrics, Research Unit, Ljubljana, Slovenia

[2] University of Ljubljana, Faculty of Pharmacy, Department of Clinical Biochemistry, Ljubljana, Slovenia

[3] University of Ljubljana, Faculty of Medicine, Department of Gynaecology and Obstetrics, Ljubljana, Slovenia

[4] University Medical Centre Ljubljana, Department of Maxillofacial and Oral Surgery, Ljubljana, Slovenia

[5] University Medical Centre Ljubljana, University Children's Hospital, Clinical Institute for Special Laboratory Diagnostics, Ljubljana, Slovenia

[6] University of Ljubljana, Faculty of Medicine, Institute of Biochemistry and Molecular Genetics, Ljubljana, Slovenia

[7] University Medical Centre Ljubljana, Clinical Institute of Genomic Medicine, Ljubljana, Slovenia

***Corresponding author:**
Assoc. prof. Nataša Karas Kuželički, PhD
Tel: +386-1-47-69-629
Email: natasa.karas@ffa.uni-lj.si

Table S1. The fold change in copy number (ΔKCt) of *TBX22* coding exons calculated for the samples of family 7 (F-7) and controls

	**Fold change in copy number (ΔKCt)[a]**															
***TBX22* exon number**	**2**		**3**		**4**		**5**		**6**		**7**		**8**		**9**	
**Reference gene**	***G6PD***	***IRF6***	***G6PD***	***IRF6***	***G6PD***	***IRF6***	***G6PD***	***IRF6***	***G6PD***	***IRF6***	***G6PD***	***IRF6***	***G6PD***	***IRF6***	***G6PD***	***IRF6***
**Sample (sex)**																
**Normal controls**																
Control 1 (F)	-0.074	-0.114	-0.042	-0.080	0.006	0.090	0.221	0.184	0.002	-0.036	-0.006	0.076	-0.003	0.103	-0.093	0.063
Control 2 (F)	0.035	-0.017	-0.016	-0.066	-0.115	-0.042	0.002	-0.046	-0.095	-0.145	-0.125	-0.054	-0.159	-0.064	-0.164	-0.023
Control 3 (F)	0.107	-0.024	0.085	-0.040	-0.041	-0.048	0.126	0.004	-0.018	-0.143	-0.020	-0.023	-0.060	-0.040	-0.083	-0.040
Control 4 (F)	-0.068	0.154	-0.027	0.185	0.150	N.A.	-0.349	-0.141	0.111	0.323	0.151	N.A.	0.221	N.A.	0.341	N.A.
Control 5 (M)	0.152	0.184	-0.035	-0.006	-0.011	0.019	-0.025	0.004	0.038	0.070	-0.007	0.023	-0.017	0.014	-0.032	0.004
Control 6 (M)	-0.058	-0.066	0.045	0.038	-0.068	-0.076	0.165	0.157	-0.032	-0.040	-0.114	-0.121	-0.107	-0.115	0.033	0.024
Control 7 (M)	-0.094	-0.118	-0.010	-0.032	0.080	0.057	-0.140	-0.162	-0.007	-0.030	0.121	0.099	0.124	0.101	-0.001	-0.028
**Test samples**																
F-7 IV-2 (M)	x	x	x	x	x	x	x	x	x	x	x	x	x	x	x	x
F-7 III-4 (F)	-0.678	-0.900	-0.841	-1.053	-0.851	-0.948	-0.855	-1.063	-0.963	-1.176	-0.828	-0.918	-0.884	-0.951	-0.654	-0.650
F-7 III-2 (F)	-0.702	-1.084	-0.835	-1.200	-0.787	-1.042	-0.790	-1.147	-0.944	-1.309	-0.805	-1.046	-0.855	-1.073	-0.740	-1.006
F-7 IV-1 (M)	x	x	x	x	x	x	x	x	x	x	x	x	x	x	x	x

F, female; M, male; x, no presence of real-time quantitative PCR (qPCR) product after amplification of tested *TBX22* exon, but qPCR product present when amplifying the exon of the reference gene; N.A., data not available

[a], ΔKCt values of 0 ± 0.35 indicate no copy number change (no genetic abnormality) in males and females, whereas -1 ± 0.35 indicates loss of one allelic copy (the deletion of the *TBX22* exon) in females. For the male samples, loss of a single allelic copy was indicated when no qPCR product was present

Table S2. Rare variants identified in families with non-syndromic orofacial clefts whose involvement could not be clearly determined based on the results of *in silico* prediction tools, co-segregation analyses, and the literature

Gene (Exon)	Genomic location (hg19)	Ref>Alt	Nucleotide change[a] 'rsID' number	Amino acid change	Variant type	ACMG class	GnomAD[b]	Predicted deleterious (Y/N)[c]	Franklin's aggregated prediction	ClinVar[d]	Segregation (Y/N)[e]
DLG1 (20)	Chr3:196,793,628	ATTAGAAAGTGAAGGCACAG>A	NM_001366207.1: c.2048-22_2048-4del rs758466083	NP_001353136.1: p.?	splice-site indel	VUS (PM2)	8.92E-06	na	na	na	x
FGFR1 (4)	Chr8:38,285,913	G>GTCA	NM_023110.3: c.396_398dup rs138489552	NP_075598.2: p.Asp133dup	in-frame indel	VUS (PM2,PM4, BP6)	4.59E-04	na	na	conflict	Y & N
BMP4 (3)	Chr14:54,418,669	G>C	NM_001202.6: c.272C>G rs121912767	NP_001193.2: p.Ser91Cys	missense	VUS (PM2,PP3, BP6)	3.86E-04	Y	Deleterious	conflict	Y
JAG2 (24)	Chr14:105,611,347	T>C	NM_002226.5: c.3004A>G	NP_002217.3: p.Arg1002Gly	missense	VUS (PM2)	2.41E-05	N	Uncertain	na	Y & N
TBX22 (9)	chrX:79,286,536	G>A	NM_001109878.2: c.1489G>A rs761750322	NP_001103348.1: p.Asp497Asn	missense	VUS (PM2)	2.23E-04	N	Uncertain	na	Y

Y/N, yes (Y) or no (N); na, not available; conflict, conflicting interpretations of pathogenicity (uncertain significance, likely benign or benign); x, phenotype co-segregation could not be determined because the sample from the proband's affected family member was not available for the analysis; Y & N, variant segregates with phenotype but is also present in unaffected proband's sibling (possible reduced penetrance); Ref>Alt, reference and alternative allele, where reference allele is listed first

[a], All listed variants were found in one family and are all heterozygous, except for rs761750322 (*TBX22*), which is hemizygous; [b], The frequency of the alternative allele from the Genome Aggregation Database (GnomAD); [c], Predicted deleterious by *in silico* tools – missense SNV predicted deleterious by at least three *in silico* prediction tools (SIFT, PolyPhen-2, MutationAssessor, MutationTaster, FATHMM, CADD, MetaLR, REVEL or GERP++); [d], Interpretation in ClinVar database; [e], Variant co-segregates with disease phenotype in the family

Table S3. Rare variants identified in families with non-syndromic orofacial clefts excluded after co-segregation analysis because they do not segregate with the disease phenotype

Gene (Exon)	Genomic location (hg19)	Ref>Alt	Nucleotide change[a] 'rsID' number	Amino acid change	Variant type	ACMG class	GnomAD[b]	Predicted deleterious (Y/N)[c]	Franklin's aggregated prediction	ClinVar[d]
MTHFR (7)	chr1:11,854,790	G>A	NM_005957.5: c.1162C>T rs200138092	NP_005948.3: p.Arg388Cys	missense	Likely Pathogenic (PM2,PM5, PM1,PP3,PP2)	3.36E-04	Y	Deleterious	uncertain significance
MTHFR[#] (6)	chr1:11,855,315	C>T	NM_005957.5: c.871G>A rs771684838	NP_005948.3: p.Asp291Asn	missense	VUS (PM2,PP3, PP2)	2.75E-05	Y	Deleterious	na
TBX10 (3)	chr11:67,402,333	A>T	NM_005995.5: c.331T>A rs117056541	NP_005986.2: p.Tyr111Asn	missense	VUS (PP3,PM2, BS2)	0.004743371	Y	Deleterious	na
MMP3 (4)	chr11:102,712,927	C>A	NM_002422.5: c.583G>T rs782241657	NP_002413.1: p.Ala195Ser	missense	VUS (PM2)	1.76E-05	N	Uncertain	na
COL2A1 (44)	chr12:48,371,857	C>T	NM_001844.5: c.3047G>A rs146046296	NP_001835.3: p.Arg1016Lys	missense	VUS (PM2,PM1, PP2,BP6)	1.22E-04	Y	Uncertain	conflict
MMP25 (3)	chr16:3,100,129	C>T	NM_022468.5: c.352C>T rs149856253	NP_071913.1: p.Arg118*	nonsense	VUS (PM2)	5.70E-04	Y	na	na
RARA[#] (7)	chr17:38,510,757	A>C	NM_000964.4: c.1011A>C	NP_000955.1: p.Gly337Gly	splice-site	VUS (PM2,PP3)	na	Y	Deleterious	na
MMP9 (6)	chr20:44,640,347	T>A	NM_004994.3: c.958T>A rs202151407	NP_004985.2: p.Tyr320Asn	missense	VUS (PM2)	3.30E-05	Y	Uncertain	na
TBX22 (4)	chrX:79,279,653	A>C	NM_001109878.2: c.448A>C rs1156605251	NP_001103348.1: p.Lys150Gln	missense	VUS (PM2)	9.23E-05	Y	Uncertain	na

[#], Variant detected in proband with Pierre-Robin sequence; Y/N, yes (Y) or no (N); na, not available; conflict, conflicting interpretations of pathogenicity (uncertain significance, likely benign or benign); Ref>Alt, reference and alternative allele, where reference allele is listed first

[a], All listed variants are heterozygous and were all found in one family, with the exception of rs117056541 (*TBX10*), which was identified in two families; [b], The frequency of the alternative allele from the Genome Aggregation Database (GnomAD); [c], Predicted deleterious by *in silico* tools – missense SNV predicted deleterious by at least three *in silico* prediction tools (SIFT, PolyPhen-2, MutationAssessor, MutationTaster, FATHMM, CADD, MetaLR, REVEL or GERP++), splice site SNV predicted to alter splicing by at least one splice site prediction tool (MaxEntScan, dbscSNV Ada, or SpliceAI), and nonsense SNV predicted deleterious by two tools (MutationTaster and CADD); [d], Interpretation in ClinVar database

Table S4. Primer sequences and parameters used for Sanger sequencing of protein-coding exons and flanking intronic regions of *GRHL3*, *IRF6,* and *TBX22*

		Primer pairs' sequences	Genomic location (GRCh38/hg38)	Size [bp]	Ta [°C]	c Mg^{2+} [mM]	c F/R [µM]
GRHL3*							
Exon 1	F<br>R	5'-AGTTATCTGTTTGTCCCTGTACT-3'<br>5'-AGAAAGCCTCGATGGGATTG-3'	Chr1:24,319,155-24,319,912	758	58.9	2.5	0.4
Exon 1'	F<br>R	5'-ACGGAAGCACTGGGATCTTA-3'<br>5'-TTCCCCTTAACGACTCCAAA-3'	Chr1:24,322,951-24,323,187	237	56.4	2.5	0.4
Exon 2	F<br>R	5'-TTGACTCTCCTTACTTGCATTCAG-3'<br>5'-GTCCAGAGGGGGTAGACGTA-3'	Chr1:24,331,402-24,331,633	232	62.4	3.5	1.0
Exon 3	F<br>R	5'-GATTGAGGCTCCTACCAGCA-3'<br>5'-CTGTGCCTCCTGTGCCTAAT-3'	Chr1:24,334,583-24,334,807	225	62.4	2.0	0.2
Exon 4	F<br>R	5'-GATCAAAGGCCAGTGTGTCA-3'<br>5'-GTACATGGCAATCCCGTCTC-3'	Chr1:24,336,385-24,336,963	579	66.9	2.0	0.4
Exon 5	F<br>R	5'-GAGGCTTCCCAGAGTGAATG-3'<br>5'-TGATTGTGCCTGCTATGCTC-3'	Chr1:24,337,021-24,337,248	228	62.4	2.0	0.4
Exon 6	F<br>R	5'-TGGTTTTGTCACCCCTCAAG-3'<br>5'-GCCTCTTCCTAGAGCCTGGT-3'	Chr1:24,337,388-24,337,960	573	62.4	2.0	0.2
Exon 7	F<br>R	5'-CATTGGAACCAGGCTCTAGG-3'<br>5'-CTGGAATGGAGGCTTGAGAA-3'	Chr1:24,337,934-24,338,132	199	62.4	2.0	0.4
Exon 8	F<br>R	5'-CTCCTTCTGGTCTCCTGTGG-3'<br>5'-CGTCGGAGCAAATGACACTA-3'	Chr1:24,339,642-24,339,866	225	62.4	2.0	0.4
Exon 9	F<br>R	5'-TGTACCAACAGCGGTGAAAA-3'<br>5'-GTCAGGGTTTGGCCCTTCTA-3'	Chr1:24,341,837-24,342,334	498	62.4	2.0	0.2
Exon 10	F<br>R	5'-CTTCTGTCTGTCCGCCTCTC-3'<br>5'-GCCGAAGGTAGGTCGTCTC-3'	Chr1:24,342,464-24,342,948	485	62.4	2.0	0.2
Exon 11	F<br>R	5'-GTGAAGGGGAGAAGGAGACC-3'<br>5'-ACAGTGAGCACCACAAGTGC-3'	Chr1:24,342,811-24,343,355	545	66.9	2.0	0.2
Exon 12	F<br>R	5'-TCCACTCAACATCTCAGGTCA-3'<br>5'-AGGGAGGCGTGTGGTTGT-3'	Chr1:24,344,716-24,344,961	246	62.4	1.0	0.4
Exon 13	F<br>R	5'-GCATCCACCCTTGTGTTTTC-3'<br>5'-GTGGGCCTGGTGAGTAAATG-3'	Chr1:24,346,437-24,346,679	243	62.4	2.0	0.4
Exon 14	F<br>R	5'-CCCCTGAGATGATCCTGTTC-3'<br>5'-CCTCCTGTGGGAAATCAGAA-3'	Chr1:24,347,407-24,347,646	240	62.4	2.5	0.4
Exon 15	F<br>R	5'-CAGCAGGCAATGAATCACC-3'<br>5'-GCAGATGGAGAGGTGACCAG-3'	Chr1:24,350,023-24,350,269	247	62.4	1.0	0.4
Exon 16	F<br>R	5'-GTGCCTAAAATGTGGCAGGT-3'<br>5'-CTGACCAAAGGTTGAGAAATCC-3'	Chr1:24,354,257-24,354,938	682	62.4	2.0	0.2
Exon 16'	F<br>R	5'-ACTTTCCCTGCAAACTCGAA-3'<br>5'-CCTTCCCTTAAAGAGCTAGGC-3'	Chr1:24,364,134-24,364,529	396	65.1	2.0	0.4
IRF6							
Exon 3	F<br>R	5'-TCTAGTAGATGGGAAAGGTGGC-3'<br>5'-AGCATTCTCTCTGTTTCACCAG-3'	Chr1:209,801,133 - 209,801,505	373	62.4	2.0	0.4
Exon 4	F<br>R	5'-TCTGTAAATCGGGGTTGGGA-3'<br>5'-AGGCTTTCTTGCTTTATCCATCT-3'	Chr1:209,796,223 - 209,796,720	498	62.4	2.0	0.4
Exon 5	F<br>R	5'-TGTACTGAACCTGAGGAGCC-3'<br>5'-TCACCTCTGACTCCCACTTG-3'	Chr1:209,795,136 - 209,795,534	399	62.4	2.0	1.0
Exon 6	F<br>R	5'-CTGATGGGCTGGAAGATCAC-3'<br>5'-GCTAGCCAGGAAACAGAAACA-3'	Chr1:209,792,125 - 209,792,564	440	58.9	2.0	0.2
Exon 7	F<br>R	5'-CTGGTTGAAAGGTGGCTTGA-3'<br>5'-TTTGCCATGCCAGGAAAGC-3'	Chr1:209,790,393-209,790,989	597	62.4	2.0	0.4
Exon 8	F<br>R	5'-TCAATGATGTCCAAGAGAACCC-3'<br>5'-GGGCTGATGGATGCTTGATG-3'	Chr1:209,789,571-209,790,041	471	65.1	2.0	0.2
Exon 9	F<br>R	5'-ACAGTGAGCCTTGGCAGAA-3'<br>5'-AGCAAAGTCTGAAGGGTGATTT-3'	Chr1:209,788,234-209,788,820	587	65.1	2.0	1.0
TBX22							
Exon 2	F<br>R	5'-CTCCCTCCCTAACCCAGTTC-3'<br>5'-AGAGCTTTCCGCGAAGGTAG-3'	ChrX:80,022,206-80,022,701	496	65.1	2.5	0.2

Exon 3	F	5'-AGTGGGCATGTGAACTGTGA-3'	ChrX:80,023,001-	395	58.9	2.5	0.2
	R	5'-TGAGGGGCTATGGCTTTCTA-3'	80,023,395				
Exon 4	F	5'-GGGTATTGTGAGTCCCTTCA-3'	ChrX:80,023,951-	250	58.9	2.5	0.4
	R	5'-CCTGGGCATAGAGTACCATTC-3'	80,024,200				
Exon 5	F	5'-CTGCACCTAATGCCACAGC-3'	ChrX:80,025,570-	238	66.9	2.0	0.8
	R	5'-TCCTCGGGGTAAAGGATTGT-3'	80,025,807				
Exons 6	F	5'-GGAACTAGGGTTTGGGACTGA-3'	ChrX:80,026,644-	700	65.1	2.0	0.4
and 7	R	5'-GATGTCATTGCTATGCTGCTT-3'	80,027,343				
Exon 8	F	5'-TGGGGATGCTGAAAGTTGA-3'	ChrX:80,027,959-	247	54.4	2.0	0.2
	R	5'-CGCAGTTACCCATAAGTACACATT-3'	80,028,205				
Exon 9	F	5'-GCAGGAACATCAAATGTCAAG-3'	ChrX:80,030,444-	651	58.9	2.5	0.4
	R	5'-TTGCTGGATACCAATGAACA-3'	80,031,094				

*, *GRHL3* exons listed based on the canonical transcript (alternative exons found in other transcipts are marked with »’«)

Size, amplicon size; Ta, optimal annealing temperature; c Mg^{2+}, optimal Mg^{2+} concentration; c F/R, optimal primer concentration

Table S5. 72 additional genes used for the second step of the sequence analysis

Gene symbol	Gene name
ABCA4	ATP Binding Cassette Subfamily A Member 4
ADH1C	Alcohol Dehydrogenase 1C (Class I), Gamma Polypeptide
APC	APC Regulator Of WNT Signaling Pathway
ARHGAP29	Rho GTPase Activating Protein 29
ASS1	Argininosuccinate Synthase 1
AXIN2	Axin 2
BCL3	BCL3 Transcription Coactivator
BHMT	Betaine--Homocysteine S-Methyltransferase
BMP2	Bone Morphogenetic Protein 2
BMP4	Bone Morphogenetic Protein 4
BRIP1	BRCA1 Interacting Helicase 1
BSPRY	B-Box And SPRY Domain Containing
CCL2	C-C Motif Chemokine Ligand 2
CDH1	Cadherin 1
CLPTM1	CLPTM1 Regulator Of GABA Type A Receptor Forward Trafficking
COL2A1	Collagen Type II Alpha 1 Chain
COL11A2	Collagen Type XI Alpha 2 Chain
CTNNB1	Catenin Beta 1
DHFR	Dihydrofolate Reductase
DLG1	Discs Large MAGUK Scaffold Protein 1
DVL2	Dishevelled Segment Polarity Protein 2
EDN1	Endothelin 1
FGF1	Fibroblast Growth Factor 1
FGF2	Fibroblast Growth Factor 2
FGF3	Fibroblast Growth Factor 3
FGF10	Fibroblast Growth Factor 10
FGFR1	Fibroblast Growth Factor Receptor 1
FN1	Fibronectin 1
FOXE1	Forkhead Box E1
GCH1	GTP Cyclohydrolase 1
GNMT	Glycine N-Methyltransferase
GREM1	Gremlin 1, DAN Family BMP Antagonist
GSK3B	Glycogen Synthase Kinase 3 Beta
JAG2	Jagged Canonical Notch Ligand 2
MAFB	MAF BZIP Transcription Factor B
MMP3	Matrix Metallopeptidase 3
MMP9	Matrix Metallopeptidase 9
MMP13	Matrix Metallopeptidase 13
MMP25	Matrix Metallopeptidase 25
MSX1	Msh Homeobox 1
MTHFD1	Methylenetetrahydrofolate Dehydrogenase, Cyclohydrolase And Formyltetrahydrofolate Synthetase 1
MTHFR	Methylenetetrahydrofolate Reductase
MTR	5-Methyltetrahydrofolate-Homocysteine Methyltransferase
MTRR	5-Methyltetrahydrofolate-Homocysteine Methyltransferase Reductase
NECTIN1	Nectin Cell Adhesion Molecule 1
NECTIN2	Nectin Cell Adhesion Molecule 2
NOG	Noggin

PAH	Phenylalanine Hydroxylase
PCYT1A	Phosphate Cytidylyltransferase 1A, Choline
PIAS1	Protein Inhibitor Of Activated STAT 1
PIAS2	Protein Inhibitor Of Activated STAT 2
PON1	Paraoxonase 1
PTCH1	Patched 1
PVR	PVR Cell Adhesion Molecule
RARA	Retinoic Acid Receptor Alpha
RING1	Ring Finger Protein 1
SKI	SKI Proto-Oncogene
SUMO1	Small Ubiquitin Like Modifier 1
SUMO3	Small Ubiquitin Like Modifier 3
TBX10	T-Box Transcription Factor 10
TCN2	Transcobalamin 2
TGFA	Transforming Growth Factor Alpha
TGFB1	Transforming Growth Factor Beta 1
TGFB3	Transforming Growth Factor Beta 3
TIMP2	TIMP Metallopeptidase Inhibitor 2
TIMP3	TIMP Metallopeptidase Inhibitor 3
VAX1	Ventral Anterior Homeobox 1
WNT3	Wnt Family Member 3
WNT3A	Wnt Family Member 3A
WNT5A	Wnt Family Member 5A
WNT8A	Wnt Family Member 8A
WNT9B	Wnt Family Member 9B

Table S6. Primer sequences and parameters used for variant validation and co-segregation analysis by Sanger sequencing

Gene [exon] (variant)*		Primer pairs' sequences	Genomic location (GRCh38/hg38)	Size [bp]	Ta [°C]	c Mg^{2+} [mM]	c F/R [µM]
MTHFR [7] (rs200138092)	F<br>R	5'-CGCTCCCAAGAACAAAGATG-3'<br>5'-CCCTCTAGCCAATCCCTTGT-3'	Chr1:11,794,679-11,794,912	234	56.4	2.0	0.2
MTHFR [6] (rs771684838)	F<br>R	5'-ATGGCACAGACATCCATCTG-3'<br>5'-CCCAGGTGTGCTCCTAGAAT-3'	Chr1:11,794,999-11,795,495	497	62.4	2.0	0.2
GRHL3 [10] (c.1285G>T)	F<br>R	5'-CTTCTGTCTGTCCGCCTCTC-3'<br>5'-GCCGAAGGTAGGTCGTCTC-3'	Chr1:24,342,464-24,342,948	485	62.4	2.0	0.2
IRF6 [3] (rs121434229)	F<br>R	5'-TCTAGTAGATGGGAAAGGTGGC-3'<br>5'-AGCATTCTCTCTGTTTCACCAG-3'	Chr1:209,801,133-209,801,505	373	62.4	2.0	0.4
IRF6 [6] (c.622C>T)	F<br>R	5'-CTGATGGGCTGGAAGATCAC-3'<br>5'-GCTAGCCAGGAAACAGAAACA-3'	Chr1:209,792,125-209,792,564	440	58.9	2.0	0.2
IRF6 [7] (c.687delG)	F<br>R	5'-CTGGTTGAAAGGTGGCTTGA-3'<br>5'-TTTGCCATGCCAGGAAAGC-3'	Chr1:209,790,393-209,790,989	597	62.4	2.0	0.4
IRF6 [9] (rs1553247595)	F<br>R	5'-ACAGTGAGCCTTGGCAGAA-3'<br>5'-AGCAAAGTCTGAAGGGTGATTT-3'	Chr1:209,788,234-209,788,820	587	65.1	2.0	1.0
DLG1 [20] (rs758466083)	F<br>R	5'-GGCTTCACAAACCTTCTTGTT-3'<br>5'-GGAGTAGCACATAGCATCACAAA-3'	Chr3:197,066,692-197,067,041	350	62.4	2.0	0.2
FGFR1 [4] (rs138489552)	F<br>R	5'-GGAGGTGTCTTGCCCATCT-3'<br>5'-GAGGGTGTCCGTGTTCATCT-3'	Chr8:38,428,177-38,428,527	351	62.4	2.0	0.2
TBX10 [3] (rs117056541)	F<br>R	5'-CCCGCACCTGTATCTCTTGT-3'<br>5'-GAAGCCTCTGTGGGAGGAAT-3'	Chr11:67,634,809-67,635,058	250	62.4	2.0	0.2
MMP3 [4] (rs782241657)	F<br>R	5'-CCCCTAGATCACAAAGATCCA-3'<br>5'-ACTGGAAAAGGCTCAAAAGG-3'	Chr11:102,841,900-102,842,342	443	62.4	2.0	0.2
COL2A1 [44] (rs146046296)	F<br>R	5'-GAAGCAGCCCTTGGTCTCTA-3'<br>5'-GTCGGTGAGTGTGGGGTATC-3'	Chr12:47,977,813-47,978,294	482	66.9	2.0	0.2
BMP4 [3] (rs121912767)	F<br>R	5'-ACTGGGGGAAGAGACTGACC-3'<br>5'-GACTTCGAGGCGACACTTCT-3'	Chr14:53,951,834-53,952,057	224	62.4	1.0	0.4
JAG2 [24] (c.3004A>G)	F<br>R	5'-CCAGGGATGTAGGTGTCAGG-3'<br>5'-GAGCCTCTGTCCAGCATAGC-3'	Chr14:105,144,848-105,145,124	277	65.1	2.0	0.2
MMP25 [3] (rs149856253)	F<br>R	5'-GCAGGCTCTCCTATTGTGGA-3'<br>5'-GGCAAAGTCGATGAGGATGT-3'	Chr16:3,049,958-3,050,410	453	62.4	2.0	0.2
RARA [7] (c.1011A>C)	F<br>R	5'-AGAGTGGGTTCGGGTTCAGT-3'<br>5'-CCCTTGCTGTGTAGTTGCAG-3'	Chr17:40,354,253-40,354,644	392	66.9	2.0	0.2
MMP9 [6] (rs202151407)	F<br>R	5'-ATGAGAGATGGGATGAACTGC-3'<br>5'-CAAACTGATGGGAGGGAAGA-3'	Chr20:46,011,460-46,011,834	375	65.1	2.0	0.2
TBX22 [4] (rs1156605251)	F<br>R	5'-GGGTATTGTGAGTCCCTTCA-3'<br>5'-CCTGGGCATAGAGTACCATTC-3'	ChrX:80,023,951-80,024,200	250	58.9	2.5	0.4
TBX22 [9] (rs761750322)	F<br>R	5'-GCAGGAACATCAAATGTCAAG-3'<br>5'-TTGCTGGATACCAATGAACA-3'	ChrX:80,030,444-80,031,094	651	58.9	2.5	0.4

*, Variants without 'rsID' number were named by the position of the nucleotide change, determined based on the Ensembl canonical transcripts

Size, amplicon size; Ta, optimal annealing temperature; c Mg^{2+}, optimal Mg^{2+} concentration; c F/R, optimal primer concentration

Table S7. Primer sequences and parameters used for real-time quantitative PCR analysis of *TBX22* coding exons

		Primer pairs' sequences	Genomic location (GRCh38/hg38)	Amplicon size (bp)	Ta (°C)	c F/R (nM)
TBX22						
Exon 2	F	5'-CTCCCTCCCTAACCCAGTTC-3'	ChrX:80,022,206-	496	64	100
	R	5'-AGAGCTTTCCGCGAAGGTAG-3'	80,022,701			
Exon 3	F	5'-CGGAGCTTGGGGAATCTG-3'	ChrX:80,022,922-	496	64	400
	R	5'-GGTAAGTCCGAGCCCTATCA-3'	80,023,417			
Exon 4	F	5'-GGGTATTGTGAGTCCCTTCA-3'	ChrX:80,023,951-	250	59	400
	R	5'-CCTGGGCATAGAGTACCATTC-3'	80,024,200			
Exon 5	F	5'-CTGCACCTAATGCCACAGC-3'	ChrX:80,025,570-	238	64	100
	R	5'-TCCTCGGGGTAAAGGATTGT-3'	80,025,807			
Exon 6	F	5'-TGGGACTGAAGCCAGTTTTT-3'	ChrX:80,026,656-	245	60	200
	R	5'-TTTCCATTCGCCTGAGACAT-3'	80,026,900			
Exon 7	F	5'-AACGTGTTTCAACATCTCTTCTGG-3'	ChrX:80,027,149-	200	60	200
	R	5'-TATTAGATGTCATTGCTATGCTGC-3'	80,027,348			
Exon 8	F	5'-GAAATTGCATTCTGGGGATG-3'	ChrX:80,027,947-	196	60	200
	R	5'-AAGGGTTCCGGATGTTGTTA-3'	80,028,142			
Exon 9	F	5'-GCAGGAACATCAAATGTCAAG-3'	ChrX:80,030,444-	651	59	400
	R	5'-TTGCTGGATACCAATGAACA-3'	80,031,094			
G6PD						
Exon 3	F	5'-TCGGGGCTCTTCTGTCTGTA-3'	ChrX:154,536,002-	331	64	400
	R	5'-CGATGAAGGTGTTTTCGGGC-3'	154,536,332			
IRF6						
Exon 5	F	5'-TGTACTGAACCTGAGGAGCC-3'	Chr1:209,795,136-	399	64	100
	R	5'-TCACCTCTGACTCCCACTTG-3'	209,795,534			

Ta, optimal annealing temperature; c F/R, optimal primer concentration

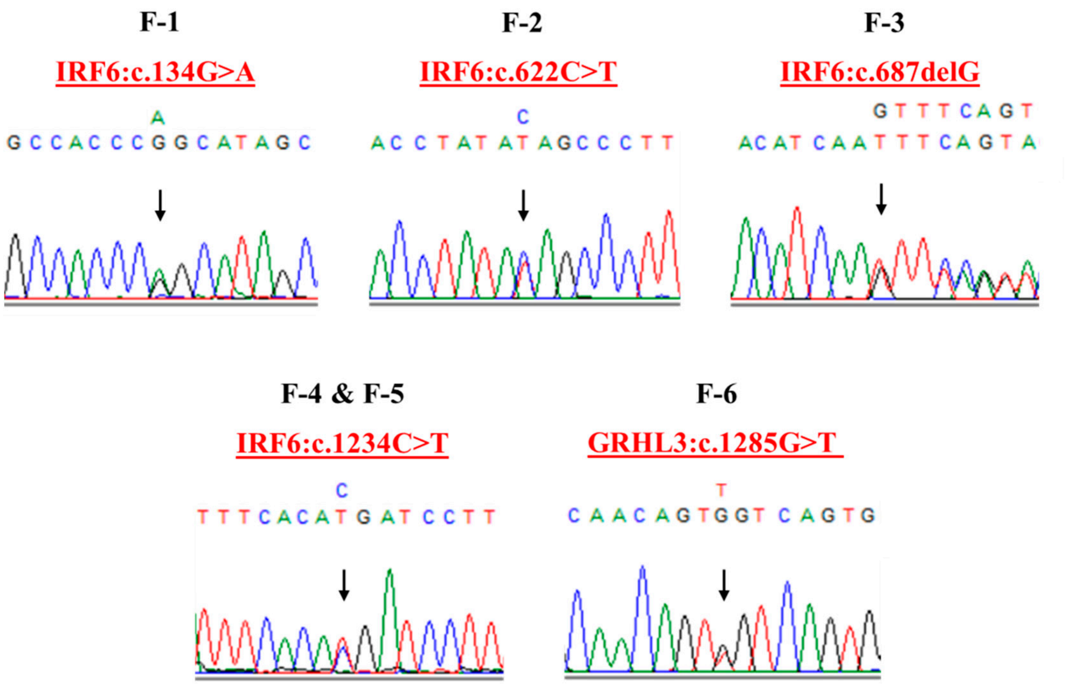

Figure S1. Sequencing chromatograms of *IRF6* and *GRHL3* variants confirming the diagnosis of Van der Woude syndrome in six families (F-1 to F-6). Each base affected by a variant is marked with an arrowhead.

A
B
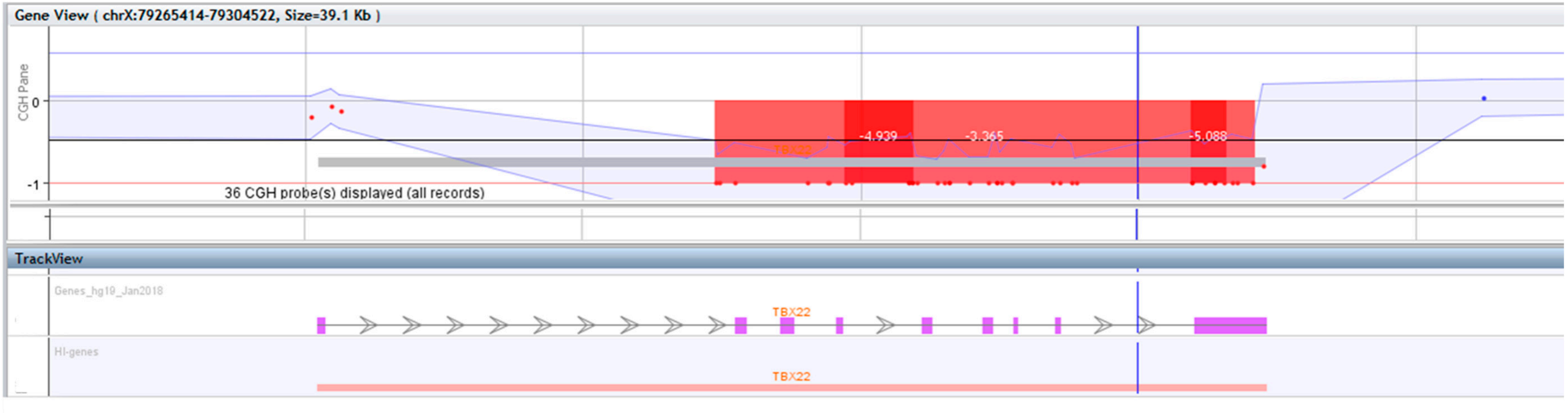

Figure S2. The result of microarray-based comparative genomic hybridization (array CGH) analysis in the proband of family 7 (F-7) with X-linked cleft palate with or without ankyloglossia. It shows the deletion (arr[GRCh37] Xq21.1(79,277,377_79,287,288)x0) encompassing exons 2-9 of the *TBX22* gene. (A), The $\log_2$ ratio values of the probes covering the region of the *TBX22* gene, with the red area depicting the deleted region. To the left and right of the deleted region are the adjacent signals with normal copy number values; (B), The canonical transcript of the gene in the deleted region, the *TBX22* gene, with the purple squares representing exons and the flanking grey lines representing introns; the pink line shows that the *TBX22* gene is haploinsufficient.
